# Supplementary material for: Predictive modelling of a novel anti-adhesion therapy to combat bacterial colonisation of burn wounds
Source: PLoS Comput Biol. 2018 May 3;14(5):e1006071. doi: 10.1371/journal.pcbi.1006071 (PMC5933687; doi:10.1371/journal.pcbi.1006071)
Supplement: S1 Supporting Information — (PDF) [file pcbi.1006071.s001.pdf]

Predictive modelling of a novel anti-adhesion therapy to combat bacterial colonisation of burn wounds
PLOS Computational Biology
S1 Supporting Information

Paul A. Roberts*[1,2], Ryan M. Huebinger[3], Emma Keen[2], Anne-Marie Krachler[4] and Sara Jabbari[1,2]

[1]School of Mathematics, University of Birmingham, Edgbaston, Birmingham, United Kingdom
[2]Institute of Microbiology and Infection, School of Biosciences, University of Birmingham, Edgbaston, Birmingham, United Kingdom
[3]Department of Surgery, University of Texas Southwestern Medical Center, Dallas, Texas, United States of America
[4]Department of Microbiology and Molecular Genetics, University of Texas McGovern Medical School at Houston, Houston, Texas, United States of America

Parameter fitting methods

For parameter sets 1, 2 and 12, the models with and without a single inhibitor dose were fitted to the respective experimental data simultaneously using MCMC, while, for the remaining parameter sets, fitting was performed first for the untreated scenario, using MCMC to determine the first 10 parameters in Table 1, and Table A in S2 Supporting Information, and then for the treatment scenario, using the Matlab routine `fmincon` to determine the three remaining (treatment-associated) parameters (rows 11–13 in Table 1, and Table A S2 Supporting Information). The goodness of fit (mean squared error) resulting from each of these methods is similar, neither being consistently better than the other; however, they do give rise to qualitatively different behaviours — all the fits from the first method fall into Cases A and D, while all those from the second method fall into Cases B and C.

MCMC fits were obtained using either the Metropolis-Hastings (MH) algorithm or a parallel tempering (PT) algorithm (see Table A, and see [1] for technical details). A PT algorithm was used since preliminary attempts at fitting with the MH algorithm suggested that the posterior is multimodal, with peaks that may be widely separated and narrow.

Priors were truncated to the ranges $[10^{-5}, 10]$ for r_F and r_B; $[10^4, 10^9]$ for K_F and K_B; $[10^{-12}, 1]$ for $\alpha_{Bac}, \beta_{Bac}$, α_A and β_A; $[10^{-7}, 10^{-2}]$ for δ_B; $[10^{-10}, 1]$, for η_{max}, $\tilde{\psi}_{Bac}$ and $\tilde{\psi}_A$; and $[10^2, 2.5 \times 10^6]$ for γ. These ranges were chosen based upon measured values where available (see below) and upon preliminary fits otherwise. Since, to the best of our knowledge, any order of magnitude within these ranges is equally likely, the target distribution was sampled from within $\log_{10}$ space, in which the prior distribution was assumed to be uniform, to avoid biasing the chains toward regions of parameter space with a higher order of magnitude. Sampling (in $\log_{10}$ space) was performed using a Gaussian transition kernel, with mean centred on the previous step and standard deviation, σ_k, taking a value in the range $\sigma_k \in [0.001, 0.1]$ (see Table A).

As noted above, the ranges of the priors are based partly upon parameter values available in the literature and partly upon preliminary fits. Campion *et al.* [2] measured the intrinsic growth rate of *Staphylococcus aureus* to be

*Corresponding author
E-mail address: p.a.roberts@univ.oxon.org (PAR)

Table A: Fitting procedures used for each parameter set. For a single fitting, models with and without treatment (a single inhibitor dose) are fitted to the experimental data simultaneously using MCMC, whereas, for a sequential fitting, the model without treatment is fitted first using MCMC, after which the model with treatment is fitted, using the method given in the last column, to determine the remaining parameters (associated with treatment). MH: Metropolis-Hastings, PT: parallel tempering. Num. Iter.: number of iterations. σ_k: standard deviation of the Gaussian transition kernel.

Parameter Set	Single/Sequential Fitting	MCMC Method	Num. Iter.	σ_k	Treatment Fitting Method
1	Single	MH	5.4×10^5	0.075	N/A
2	Single	MH	2.2×10^6	0.01	N/A
3	Sequential	MH	1.8×10^6	0.005	active-set
4	Sequential	MH	2.9×10^6	0.001	active-set
5	Sequential	PT	3×10^5	0.01	interior-point
6	Sequential	PT	3×10^5	0.05	interior-point
7	Sequential	MH	2.3×10^6	0.05	interior-point
8	Sequential	MH	6.1×10^4	0.1	active-set
9	Sequential	PT	3×10^5	0.0025	active-set
10	Sequential	MH	3×10^6	0.1	active-set
11	Sequential	MH	2.4×10^6	0.01	sqp
12	Single	MH	2.3×10^6	0.05	N/A

0.93–0.99 hr^{-1} in susceptible strains and 0.66–0.70 hr^{-1} in resistant strains; Maier *et al.* [3] give growth rates (fitted to a Monod model) of 0.8–1.4 hr^{-1} for *Escherichia coli* and 0.38–0.47 hr^{-1} for *P. aeruginosa*, while our preliminary fits of *in vitro* measurements with *P. aeruginosa* have shown that the intrinsic growth rate can be as low as $O(10^{-3})$. We chose the range $[10^{-5}, 10]$ so as to encompass these values, allowing for the possibility that growth could be more or less rapid in a burn wound. Campion *et al.* [2] also report carrying capacities of 1.1×10^9–1.7×10^{10} CFU cm^{-3} (in the context of an *in vitro* hollow fibre system). Preliminary fits to the experimental burn wound model considered in this paper suggest that the free and bound carrying capacities are likely to take much lower values, on the order of 10^5–10^7 CFU cm^{-3}; hence, we chose a range to include these values, taking the lower of Campion *et al.*'s [2] measurements as an upper bound. Ternent *et al.* [4] followed Smith *et al.* [5] in using a value of 10^{-5} $cell^{-1}$ hr^{-1} for the rate of phagocytosis of bacteria by neutrophils. The equivalent parameter in this paper, δ_B, has the units of hr^{-1}, accounting also for the density of neutrophils. We consider a range of possible values spanning two orders of magnitude above and below Smith *et al.*'s [5] value to allow for the effects of neutrophil density. The prior for γ is bounded above by 2.5×10^6, since it cannot exceed E_{init} (see Table 2), and bounded below by 100, since a significant number of bacteria would need to bind to the host cells before the destination of bound daughter cells would be affected. In the absence of further information, large intervals of parameters space are explored for the remaining fitted parameters.

The number of MCMC iterations used to obtain each fit varies between parameter sets (see Table A): $O(10^4)$–$O(10^6)$ iterations were used for the MH algorithm, while 3×10^5 iterations were used for the PT algorithm in all cases. The number of iterations used was determined largely by the available computational resources, with some chains taking longer to proceed through each iterative step and PT being more computationally expensive than MH.

The noise was assumed to have a Gaussian distribution, with standard deviation σ. The standard deviation was set to $\sigma = 5\times10^7$ at all data points when fitting parameter sets 3–11, and was set to equal the standard deviation at each data point in Sets 1, 2 and 12. The choice between these alternative values of σ did not have a discernible effect on the outcome of the fitting procedure.

Suitable initial values were chosen for each parameter, lying within the ranges defined above. Since the full range of parameter space is explored by the chains in most cases and since Markov chains are only influenced by the previous step, the precise choice of initial values within these ranges is unimportant.

Our PT algorithm uses 8 chains, with 'tempering parameters' $\vec{\beta} = (1, 0.74, 0.55, 0.42, 0.3, 0.2, 0.13, 0.09)$ (see Gregory [1], pg. 321, for this use of terminology; these values are taken from [6]). The parameters are chosen so that

the target distribution does not vary too greatly between adjacent chains, while including target distributions that are sufficiently flattened as to increase the likelihood that the whole of parameter space is explored.

While we are able to obtain a number of good fits using MCMC — this method being much more effective than the frequentist methods we trialled (using the Matlab routine fmincon described below), which become trapped in local minima producing poor fits — we have too few experimental data points to allow our chains to converge (12 points for 13 parameters). Thus, the target distributions produced may not be reliable guides to the posterior distribution. For this reason, we work only with the best fits and not with the target distributions.

In those cases where the Matlab routine fmincon was used to obtain a fit for the treatment parameters (α_A, β_A and $\tilde{\psi}_A$), one of three algorithms was used: active-set, interior-point or sqp. All three algorithms were applied in each case and the best fit was chosen (as judged by the mean squared error). No one algorithm was universally optimal and each produced the best fit for at least one parameter set (see Table A).

References

[1] Gregory PC. Baysian Logical Data Analysis for the Physical Sciences. CUP; 2005.

[2] Campion JJ, McNamara PJ, Evans ME. Pharmacodynamic Modeling of Ciprofloxacin Resistance in Staphylococcus aureus. Antimicrob Agents Chemother. 2005;49(1):209–219.

[3] Maier RM, Pepper IL. Chapter 3 - Bacterial Growth. In: Pepper IL, Gerba CP, Gentry TJ, editors. Environmental Microbiology (Third edition). San Diego: Academic Press; 2015. p. 37–56.

[4] Ternent L, Dyson RJ, Krachler AM, Jabbari S. Bacterial fitness shapes the population dynamics of antibiotic-resistant and -susceptible bacteria in a model of combined antibiotic and anti-virulence treatment. J Theor Biol. 2015;372:1–11.

[5] Smith AM, McCullers JA, Adler FR. Mathematical model of a three-stage innate immune response to a pneumococcal lung infection. J Theor Biol. 2011;276(1):106–116.

[6] Gregory PC. Introduction to Markov chain Monte Carlo (MCMC) and its role in modern Bayesian analysis; 2010. Available at: www.astro.ufl.edu/ eford/astrostats/Florida2Mar2010.pdf (accessed June 23, 2017).
