# Supplementary material for: Predictive modelling of a novel anti-adhesion therapy to combat bacterial colonisation of burn wounds
Source: PLoS Comput Biol. 2018 May 3;14(5):e1006071. doi: 10.1371/journal.pcbi.1006071 (PMC5933687; doi:10.1371/journal.pcbi.1006071)
Supplement: S2 Supporting Information — (PDF) [file pcbi.1006071.s002.pdf]

Predictive modelling of a novel anti-adhesion therapy to combat bacterial colonisation of burn wounds
PLOS Computational Biology
S2 Supporting Information

Paul A. Roberts*[1,2], Ryan M. Huebinger[3], Emma Keen[2], Anne-Marie Krachler[4] and Sara Jabbari[1,2]

[1]School of Mathematics, University of Birmingham, Edgbaston, Birmingham, United Kingdom
[2]Institute of Microbiology and Infection, School of Biosciences, University of Birmingham, Edgbaston, Birmingham, United Kingdom
[3]Department of Surgery, University of Texas Southwestern Medical Center, Dallas, Texas, United States of America
[4]Department of Microbiology and Molecular Genetics, University of Texas McGovern Medical School at Houston, Houston, Texas, United States of America

Data and results for all 12 parameter sets

*Corresponding author
E-mail address: p.a.roberts@univ.oxon.org (PAR)

Table A: Fitted parameter values for Eqs 1–8 and stability properties. The last three rows of the top section of the table give the bacteria association constant, α_{Bac}/β_{Bac}, the inhibitor association constant, α_A/β_A, and the ratio of bacteria to inhibitor association constants. Values are given to an accuracy of 3 significant figures. The bottom section of the table summarises the stability properties of each parameter set. Each parameter set has two steady-states in both the untreated and treated (single inhibitor dose) scenarios. The first steady-state corresponds to the absence of bacteria and can be classified as a saddle-node. Both free and bound bacteria are present at the second steady-state, which may be classified as either a stable improper node or a stable spiral. NT: no treatment. T: treatment. Node: stable improper node.

Parameter	Value											
	Case A		Case B					Case C				Case D
	Set 1	Set 2	Set 3	Set 4	Set 5	Set 6	Set 7	Set 8	Set 9	Set 10	Set 11	Set 12
r_F	7.42×10^{-2}	8.37×10^{-2}	2.10×10^{-3}	4.33×10^{-3}	3.00×10^{-3}	3.97×10^{-2}	3.30×10^{-3}	4.18×10^{-3}	4.76×10^{-5}	5.57×10^{-3}	4.28×10^{-3}	2.57×10^{-1}
r_B	8.25	1.10×10^{-1}	1.35×10^{-1}	1.45×10^{-1}	1.54×10^{-1}	1.60	2.88	1.50×10^{-1}	2.89×10^{-1}	8.81×10^{-2}	1.89×10^{-1}	5.55
K_F	1.28×10^{7}	1.17×10^{7}	1.15×10^{6}	1.91×10^{6}	3.19×10^{6}	8.23×10^{6}	3.46×10^{5}	3.12×10^{6}	1.70×10^{4}	1.95×10^{7}	3.30×10^{6}	1.85×10^{6}
K_B	8.03×10^{4}	9.96×10^{5}	1.65×10^{6}	1.62×10^{6}	1.44×10^{6}	4.15×10^{5}	6.63×10^{5}	1.48×10^{6}	1.32×10^{6}	1.79×10^{6}	1.27×10^{6}	1.43×10^{6}
α_{Bac}	4.25×10^{-11}	1.34×10^{-9}	3.09×10^{-10}	2.79×10^{-10}	2.92×10^{-10}	1.88×10^{-11}	9.19×10^{-12}	2.32×10^{-10}	1.25×10^{-10}	6.47×10^{-10}	2.03×10^{-10}	3.34×10^{-11}
β_{Bac}	8.26×10^{-1}	1.97×10^{-1}	4.11×10^{-9}	1.07×10^{-8}	1.53×10^{-8}	2.02×10^{-3}	1.09×10^{-10}	8.23×10^{-9}	3.64×10^{-10}	2.48×10^{-10}	1.38×10^{-3}	5.79×10^{-6}
δ_B	4.15×10^{-4}	1.06×10^{-3}	2.31×10^{-4}	2.37×10^{-4}	8.27×10^{-3}	1.90×10^{-6}	3.07×10^{-3}	3.97×10^{-4}	8.63×10^{-4}	2.95×10^{-5}	8.35×10^{-3}	3.02×10^{-5}
η_{max}	3.70×10^{-10}	2.95×10^{-2}	1.31×10^{-7}	2.91×10^{-7}	1.99×10^{-7}	1.23×10^{-8}	2.51×10^{-2}	3.06×10^{-7}	3.54×10^{-6}	3.37×10^{-2}	2.19×10^{-2}	1.52×10^{-2}
γ	1.23×10^{4}	3.12×10^{4}	1.83×10^{5}	6.57×10^{4}	4.66×10^{3}	1.89×10^{5}	1.36×10^{6}	3.66×10^{4}	3.15×10^{3}	1.05×10^{4}	6.67×10^{2}	1.65×10^{6}
$\tilde{\psi}_{Bac}$	1.27×10^{-1}	1.42×10^{-1}	2.13×10^{-6}	5.58×10^{-6}	1.75×10^{-5}	7.28×10^{-2}	1.96×10^{-2}	7.68×10^{-6}	3.00×10^{-5}	1.39×10^{-3}	1.17×10^{-4}	5.01×10^{-1}
α_A	1.02×10^{-5}	1.46×10^{-6}	1.56×10^{-10}	1.41×10^{-10}	1.47×10^{-10}	1.77×10^{-10}	1.12×10^{-10}	2.32×10^{-10}	1.24×10^{-10}	6.47×10^{-10}	2.04×10^{-10}	5.51×10^{-9}
β_A	4.41×10^{-12}	6.35×10^{-8}	4.11×10^{-9}	1.07×10^{-8}	1.53×10^{-8}	4.48×10^{-6}	1.09×10^{-10}	3.85×10^{-3}	3.32×10^{-3}	3.92×10^{-3}	3.33×10^{-3}	4.43×10^{-1}
$\tilde{\psi}_A$	1.51×10^{-9}	4.39×10^{-8}	2.13×10^{-6}	5.59×10^{-6}	1.76×10^{-5}	5.17×10^{-4}	4.17×10^{-7}	3.85×10^{-3}	3.35×10^{-3}	5.29×10^{-3}	2.08×10^{-3}	1.75×10^{-5}
α_{Bac}/β_{Bac}	5.14×10^{-11}	6.82×10^{-9}	7.53×10^{-2}	2.61×10^{-2}	1.91×10^{-2}	9.34×10^{-9}	8.41×10^{-2}	2.82×10^{-2}	3.43×10^{-1}	2.61	1.48×10^{-7}	5.77×10^{-6}
α_A/β_A	2.32×10^{6}	2.29×10^{1}	3.79×10^{-2}	1.32×10^{-2}	9.62×10^{-3}	3.95×10^{-5}	1.03	6.03×10^{-8}	3.74×10^{-8}	1.65×10^{-7}	6.12×10^{-8}	1.24×10^{-8}
$\frac{(\alpha_{Bac}/\beta_{Bac})}{(\alpha_A/\beta_A)}$	2.22×10^{-17}	2.97×10^{-10}	1.98	1.98	1.98	2.36×10^{-4}	8.19×10^{-2}	4.68×10^{5}	9.17×10^{6}	1.58×10^{7}	2.41	4.65×10^{2}
Steady-state	Stability-type											
NT 1	Saddle	Saddle	Saddle	Saddle	Saddle	Saddle	Saddle	Saddle	Saddle	Saddle	Saddle	Saddle
NT 2	Node	Node	Spiral	Spiral	Spiral	Node	Node	Spiral	Spiral	Spiral	Spiral	Node
T 1	Saddle	Saddle	Saddle	Saddle	Saddle	Saddle	Saddle	Saddle	Saddle	Saddle	Saddle	Saddle
T 2	Node	Node	Node	Node	Node	Node	Node	Spiral	Node	Node	Node	Node

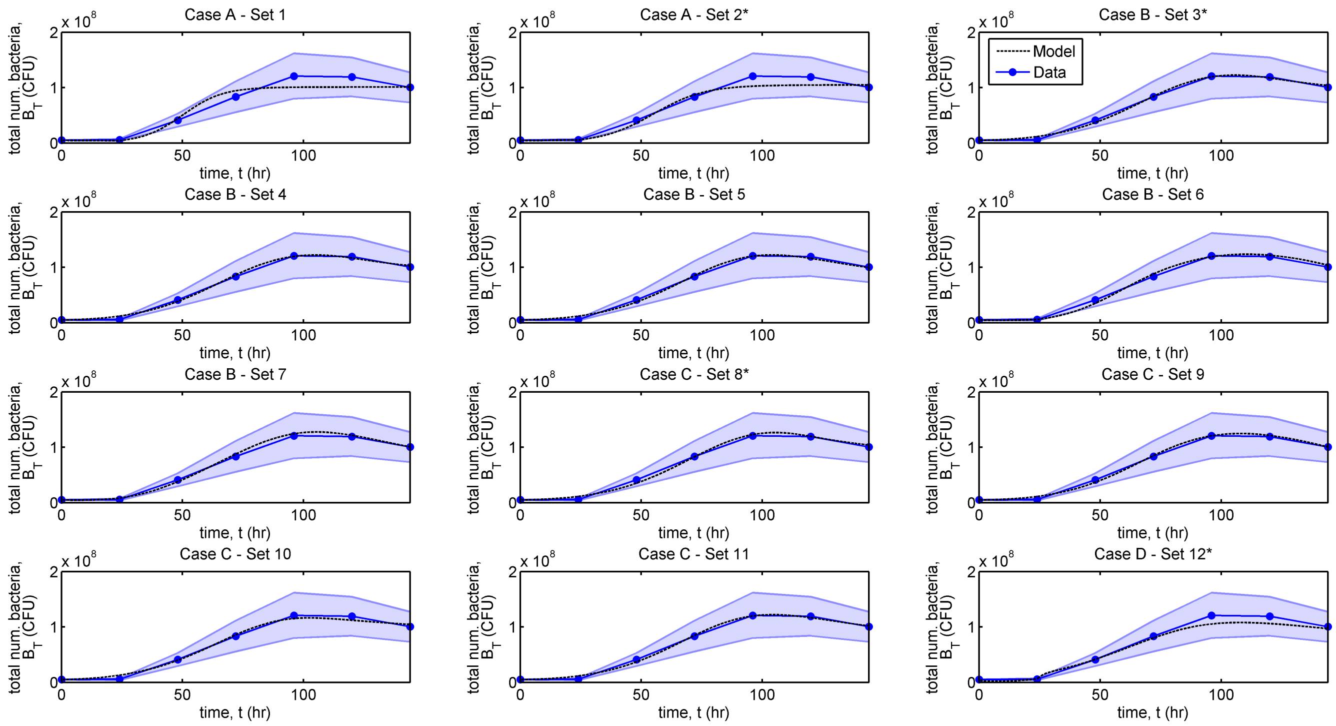

Figure A: Comparison of model predictions with experimental data in the untreated scenario. The discs mark the experimental mean, while the shaded region shows the standard error of the mean. Simulation results, denoted by the dashed line, show the total number of bacteria, $B_T(t)$ $(= VB_F(t) + A_r B_B(t))$. There is good agreement between the model and the data for all parameter sets. Graphs marked with stars are those included in the main text. Eqs 1–8 were solved using `ode15s` and fitting was performed using MCMC (see S1 Supporting Information and Table A therein). See Tables 2 and A for parameter values. CFU: colony-forming units.

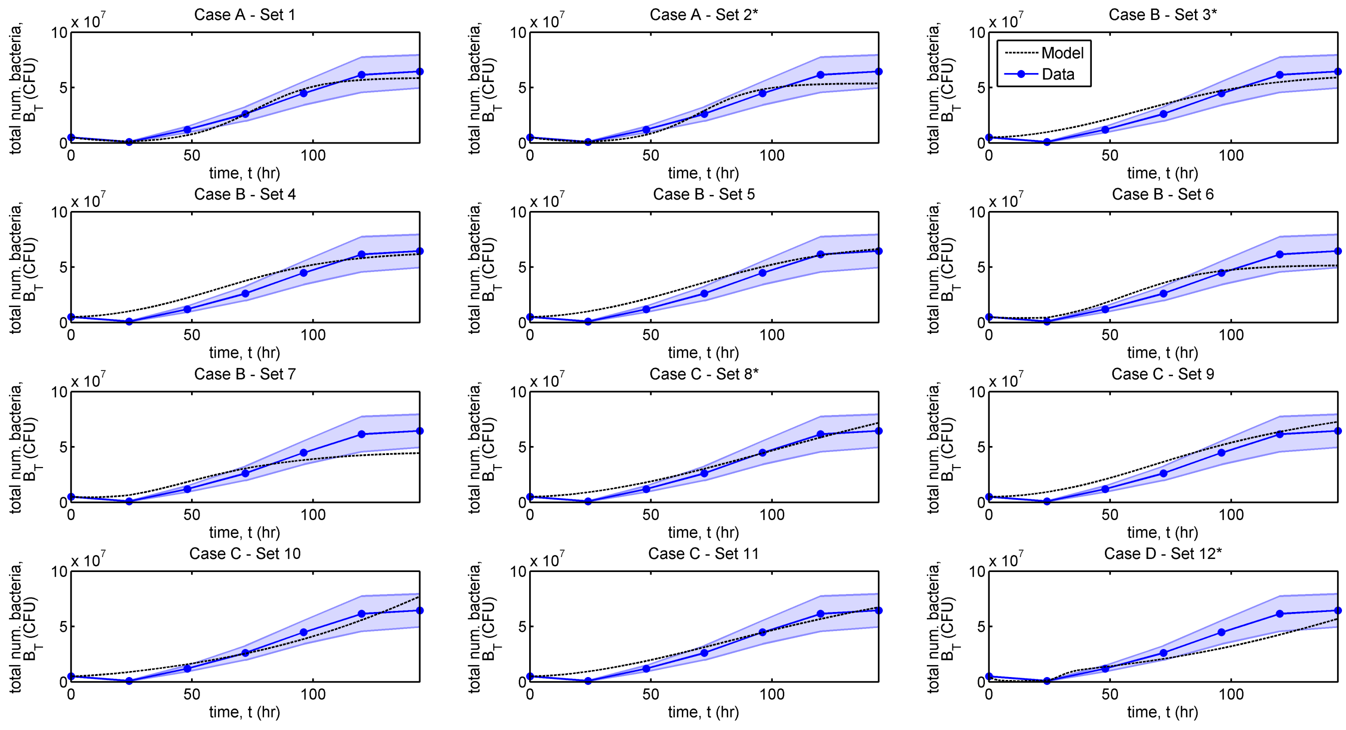

Figure B: Comparison of model predictions with experimental data in the single inhibitor dose scenario. The discs mark the experimental mean, while the shaded region shows the standard error of the mean. Simulation results, denoted by the dashed line, show the total number of bacteria, $B_T(t)$ (= $VB_F(t) + A_r B_B(t)$). There is good agreement between the model and the data for all parameter sets. Graphs marked with stars are those included in the main text. Eqs 1–8 were solved using ode15s and fitting was performed using a combination of MCMC and frequentist methods (see S1 Supporting Information and Table A therein). See Tables 2 and A for parameter values. CFU: colony-forming units.

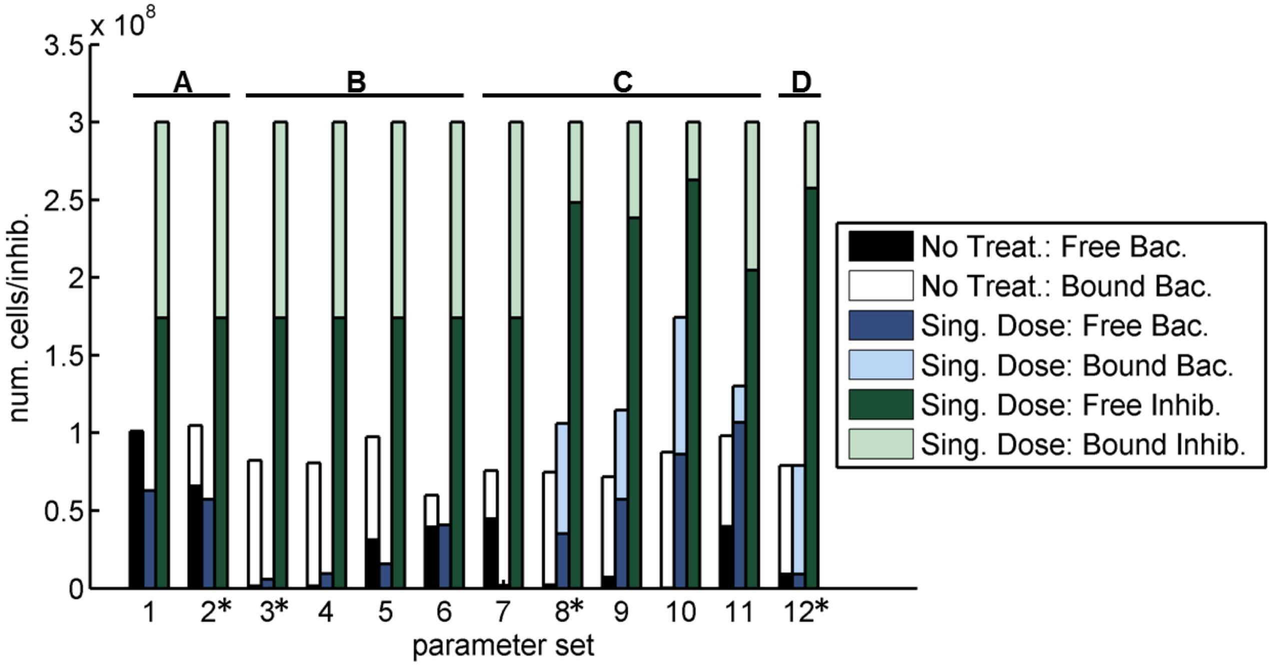

Figure C: Steady-state solutions to Eqs 1–6 with and without a single dose of inhibitor. Three stacked bars are plotted for each parameter set: the first bar shows the number of free and bound bacteria, $\hat{B}^*_{F_2} = VB^*_{F_2}$ and $\hat{B}^*_{B_2} = A_r B^*_{B_2}$, at steady-state in the untreated scenario; the second gives the number of free and bound bacteria at steady-state in the single inhibitor dose scenario, and the third shows the number of free and bound inhibitors, $\hat{A}^*_{F_2} = VA^*_{F_2}$ and $\hat{A}^*_{B_2} = A_r A^*_{B_2}$, at steady-state in the single inhibitor dose scenario. The combined height of each stacked bar gives the total number of bacteria or inhibitors, $B^*_{T_2} = \hat{B}^*_{F_2} + \hat{B}^*_{B_2}$ and $A_{F_{init}} = \hat{A}^*_{F_2} + \hat{A}^*_{B_2}$. Treatment results in a decrease in $B^*_{T_2}$ in Cases A and B, an increase in $B^*_{T_2}$ in Case C and has little effect on $B^*_{T_2}$ in Case D. The ratio of free to bound inhibitors is similar throughout Cases A and B, and varies in Cases C and D. Parameter sets marked with stars are those included in the main text. Steady-state solutions were obtained by solving Eqs 1–8 using `ode15s`, allowing the system to evolve until it reached steady-state. The problem was solved in the absence of clearance, such that $\tilde{\psi}_{Bac} = 0$ and $\tilde{\psi}_A = 0$. See Tables 2 and A for the remaining parameter values.

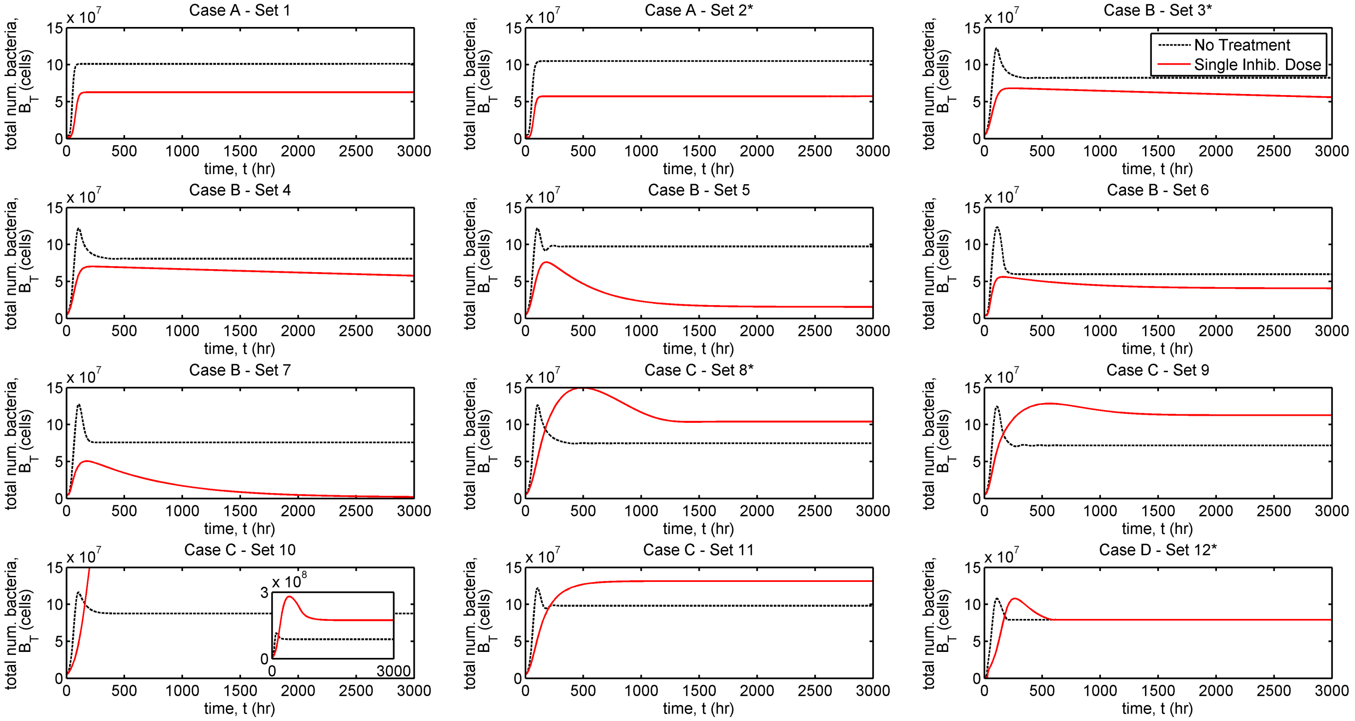

Figure D: Comparison of bacterial population dynamics in the untreated and single inhibitor dose scenarios. The total number of bacteria, $B_T(t)$ ($= VB_F(t) + A_r B_B(t)$), is plotted in each case. Simulations extend beyond the time span of the experiments, to 3000 hours = 125 days. The inset in the bottom-left panel shows the full range of the solution in the treatment scenario. Case A: the bacterial population size is an essentially monotone increasing function of time in both the untreated and single inhibitor dose scenarios, reaching an early steady-state at which the bacterial population size with treatment is roughly half that without; Case B: the bacterial population size with treatment remains below that without treatment, decreasing gradually after reaching an early maximum; Case C: treatment results in a sustained and significant increase in the bacterial population size; Case D: treatment causes the bacterial population size to temporarily exceed that without treatment, settling to a steady-state close to that of the untreated scenario. Graphs marked with stars are those included in the main text. Eqs 1–8 were solved using `ode15s`. See Tables 2 and A for parameter values.

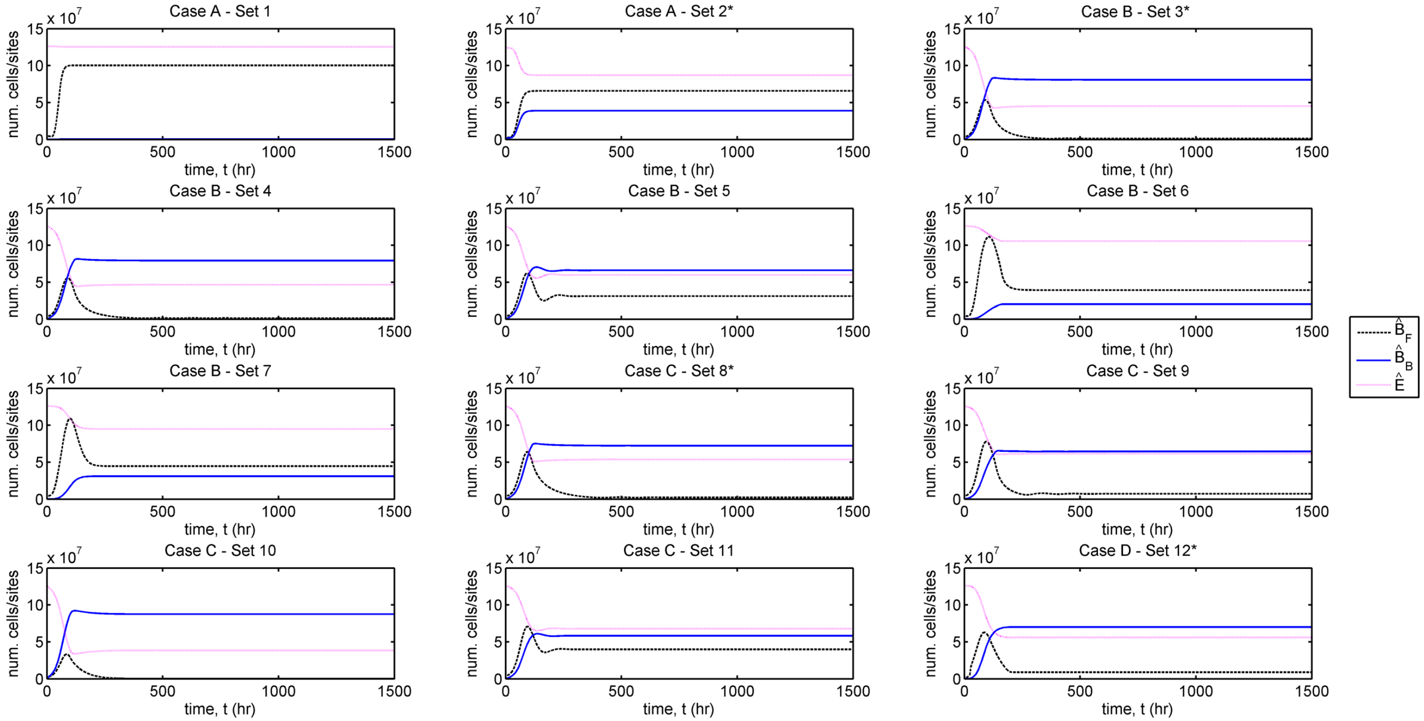

Figure E: Comparison of dependent variables in the untreated scenario. The total number of free and bound bacteria ($\hat{B}_F(t) = VB_F(t)$, $\hat{B}_B(t) = A_r B_B(t)$) and binding sites ($\hat{E}(t) = A_r E(t)$) is plotted in each case. Simulations extend beyond the time span of the experiments, to 1500 hours = 62.5 days. See Fig F legend for comparisons between the untreated and single inhibitor dose scenarios. Graphs marked with stars are those included in the main text. Eqs 1–8 were solved using `ode15s`. See Tables 2 and A for parameter values.

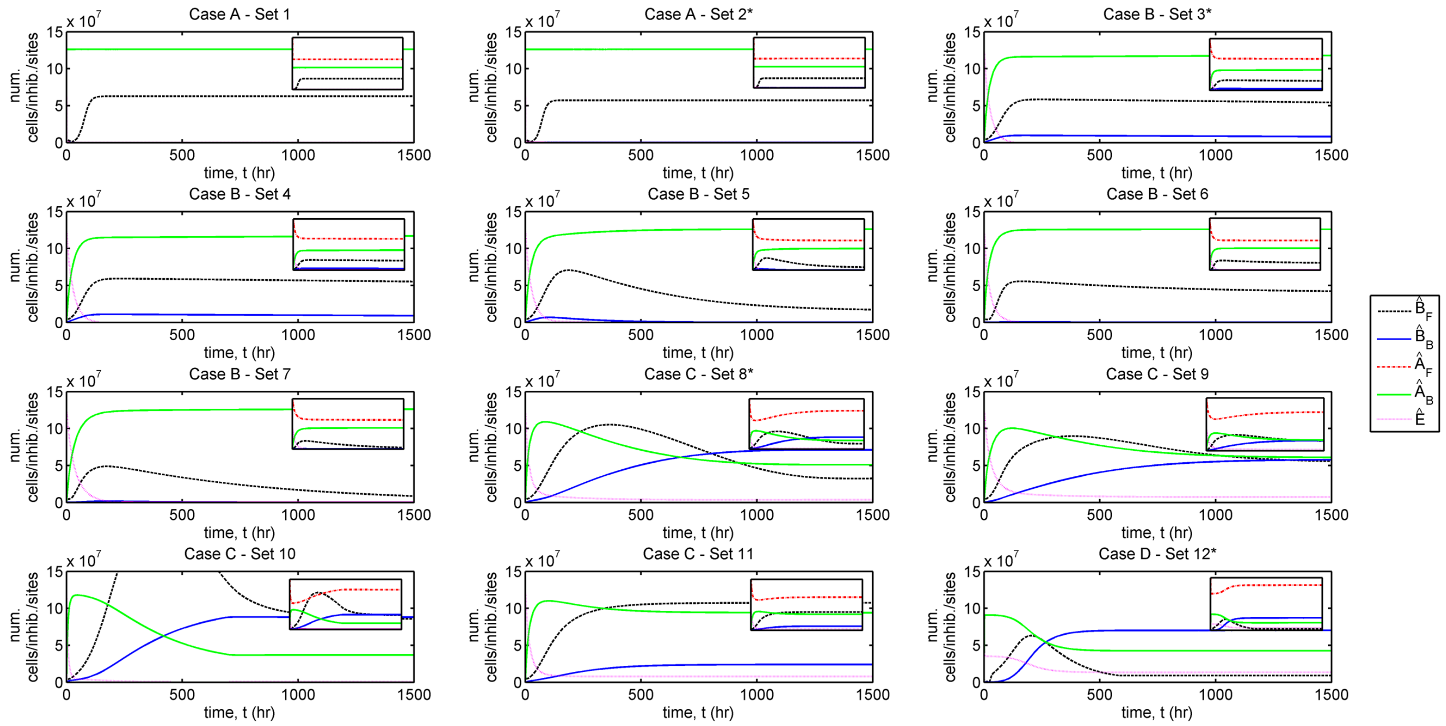

Figure F: Comparison of dependent variables in the single inhibitor dose scenario. The total number of free and bound bacteria ($\hat{B}_F(t) = VB_F(t)$, $\hat{B}_B(t) = A_rB_B(t)$), free and bound inhibitors ($\hat{A}_F(t) = VA_F(t)$, $\hat{A}_B(t) = A_rA_B(t)$) and binding sites ($\hat{E}(t) = A_rE(t)$) is plotted in each case. Simulations extend beyond the time span of the experiments, to 1500 hours = 62.5 days. Inset plots show the same simulations as in the main panels, with the y-axis in the range $y \in [0, 3\times 10^8]$. Comparing with the untreated scenario in Fig E — Cases A and B: the number of bound bacteria are reduced as a result of treatment; Case C: treatment results in an increase in the number of free bacteria; Case D: treatment increases the time taken to reach a roughly equivalent steady-state to that without treatment. Graphs marked with stars are those included in the main text. Eqs 1–8 were solved using `ode15s`. See Tables 2 and A for parameter values.

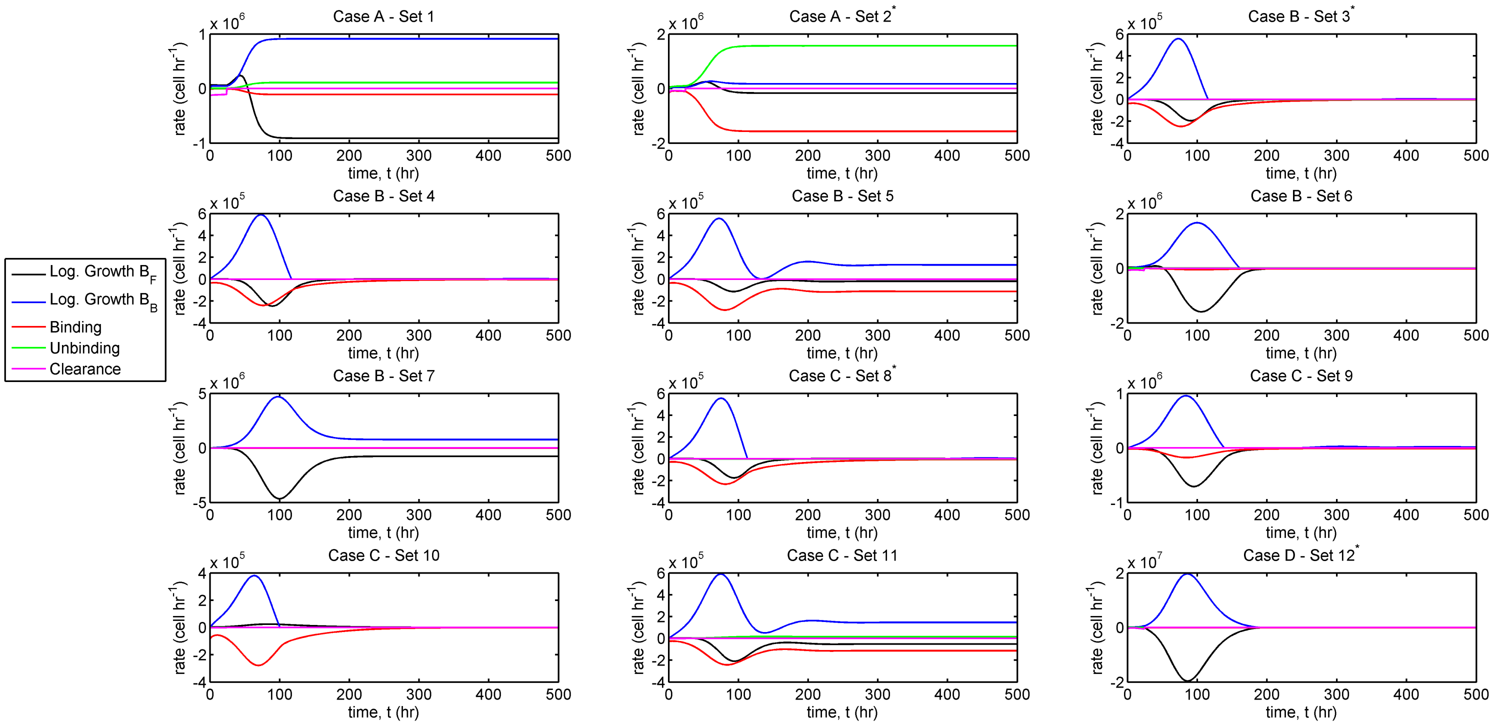

Figure G: Comparison of terms in the $\dot{B}_F$ equation (Eq 1) in the untreated scenario. Simulations extend beyond the time span of the experiments, to 500 hours ≈ 21 days. The system has settled to steady-state by $t = 500$ in all parameter sets. See Numerical solutions (main text) for discussion. Graphs marked with stars correspond to the parameter sets explored in the main text. Eqs 1–8 were solved using `ode15s`. See Tables 2 and A for parameter values.

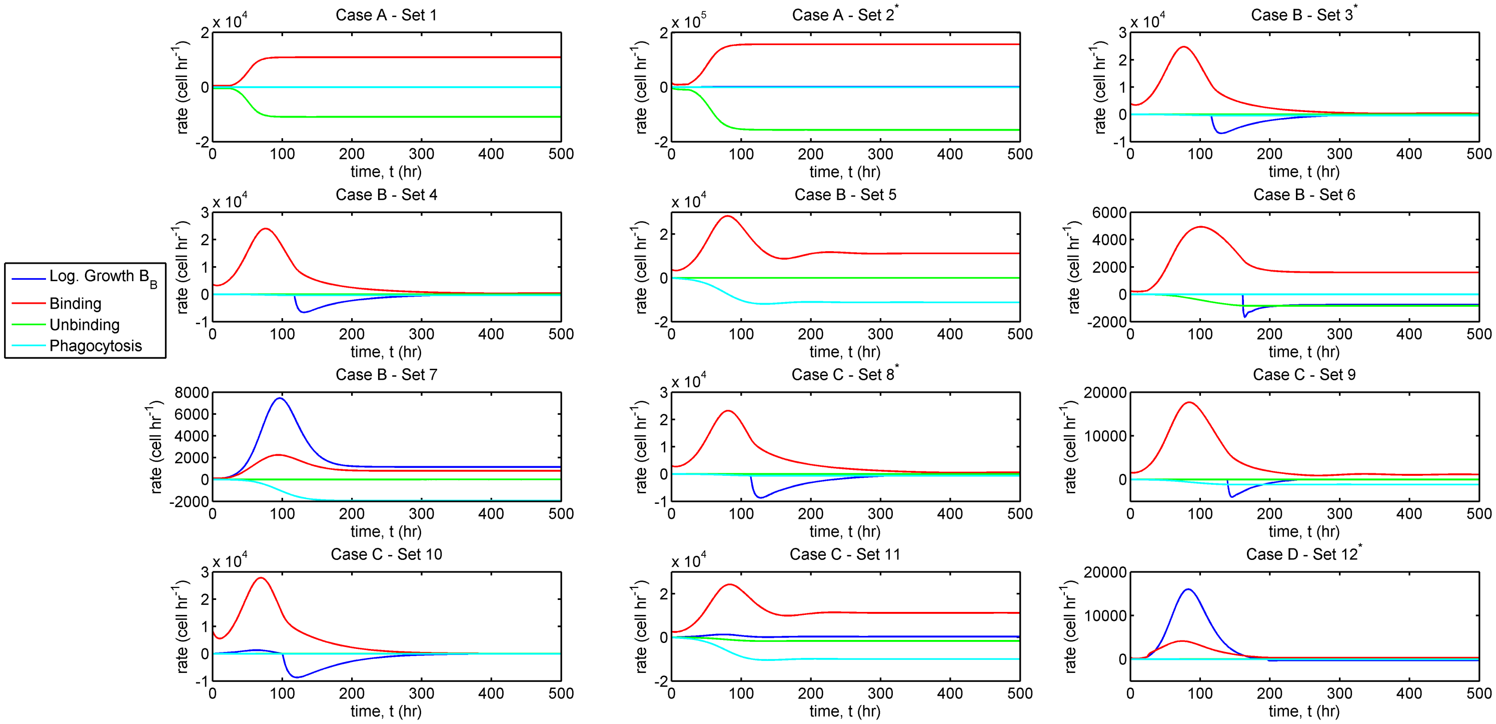

Figure H: Comparison of terms in the $\dot{B}_B$ equation (Eq 2) in the untreated scenario. Simulations extend beyond the time span of the experiments, to 500 hours ≈ 21 days. The system has settled to steady-state by $t = 500$ in all parameter sets. See Numerical solutions (main text) for discussion. Graphs marked with stars correspond to the parameter sets explored in the main text. Eqs 1–8 were solved using `ode15s`. See Tables 2 and A for parameter values.

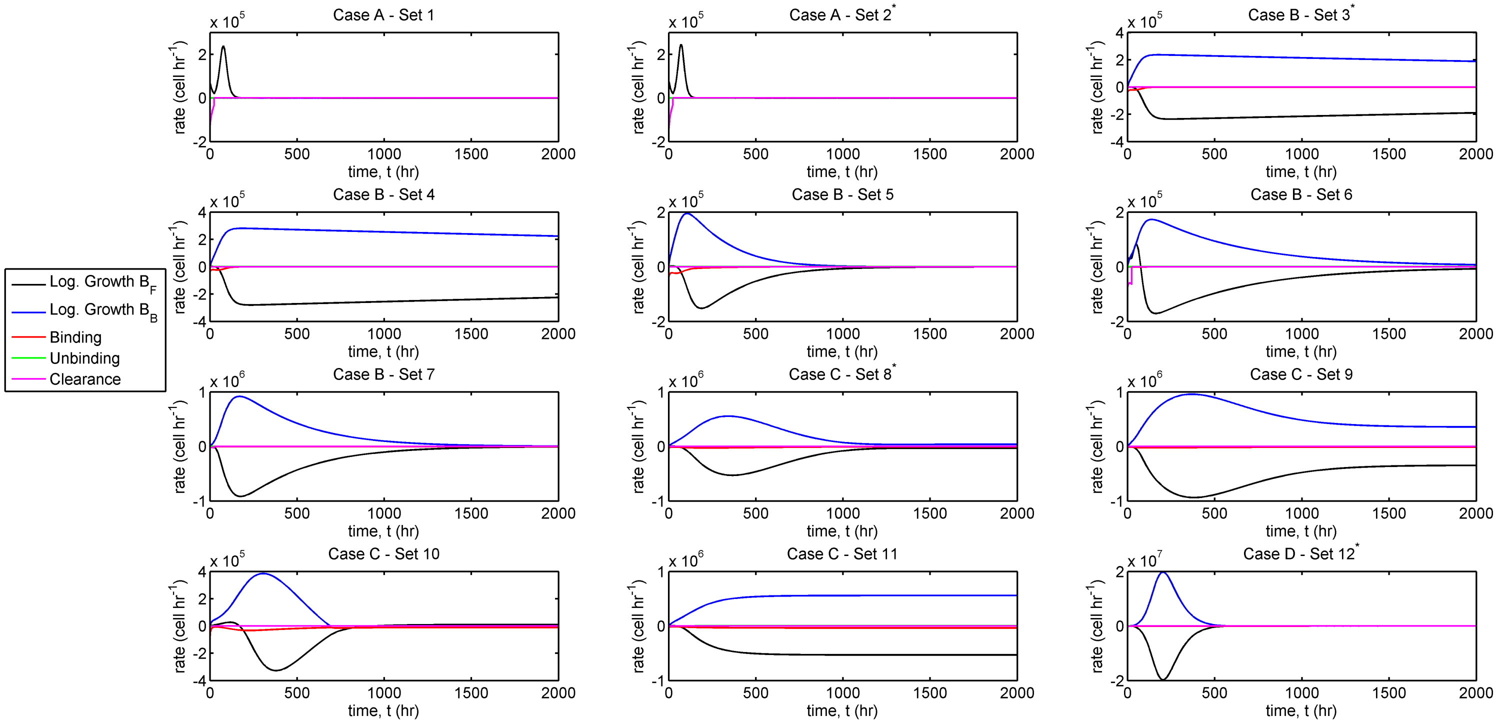

Figure I: Comparison of terms in the $\dot{B}_F$ equation (Eq 1) in the single inhibitor dose scenario. Simulations extend beyond the time span of the experiments, to 2000 hours ≈ 83 days. The system has settled to steady-state or is close to steady-state by $t = 2000$ in all parameter sets. See Numerical solutions (main text) for discussion. Graphs marked with stars correspond to the parameter sets explored in the main text. Eqs 1–8 were solved using `ode15s`. See Tables 2 and A for parameter values.

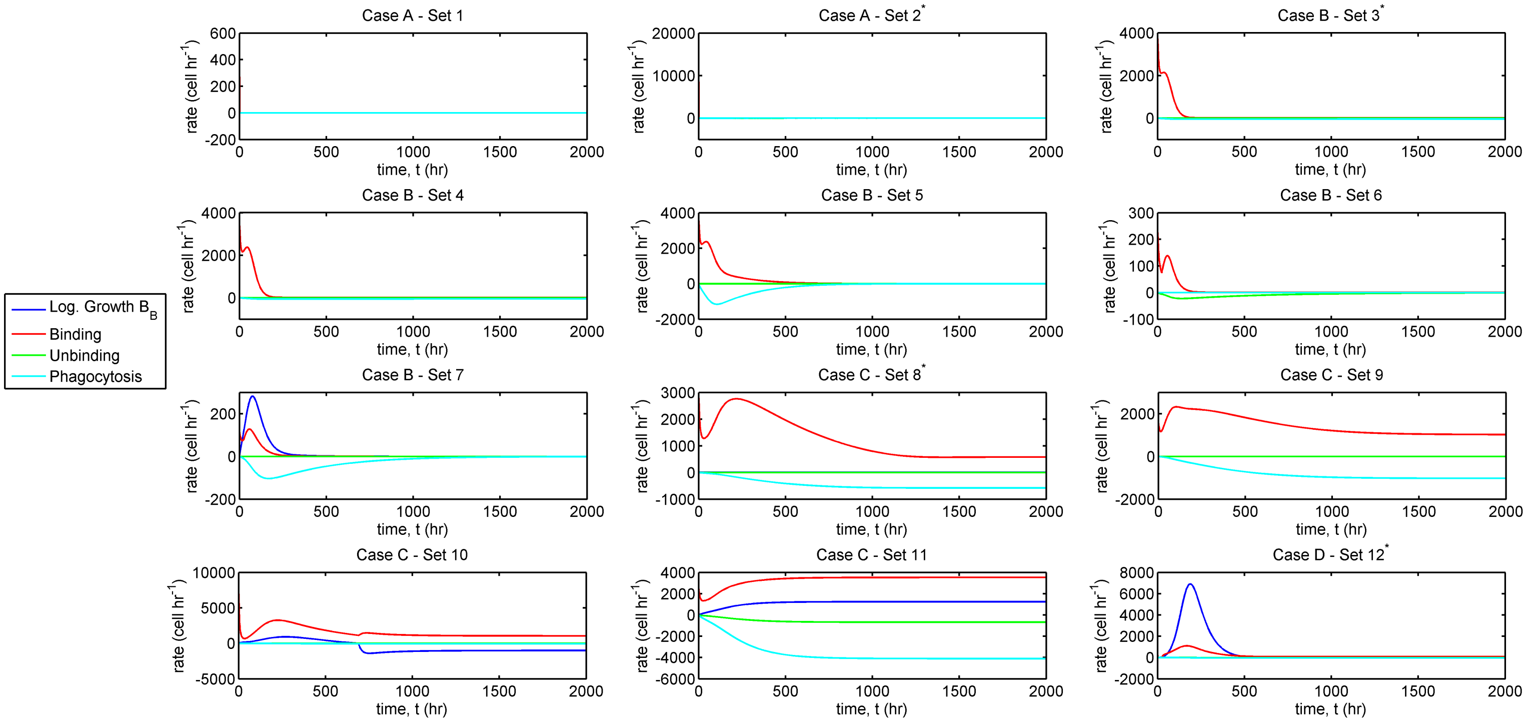

Figure J: Comparison of terms in the $\dot{B}_B$ equation (Eq 2) in the single inhibitor dose scenario. Simulations extend beyond the time span of the experiments, to 2000 hours $\approx$ 83 days. The system has settled to steady-state or is close to steady-state by $t = 2000$ in all parameter sets. See Numerical solutions (main text) for discussion. Graphs marked with stars correspond to the parameter sets explored in the main text. Eqs 1–8 were solved using `ode15s`. See Tables 2 and A for parameter values.

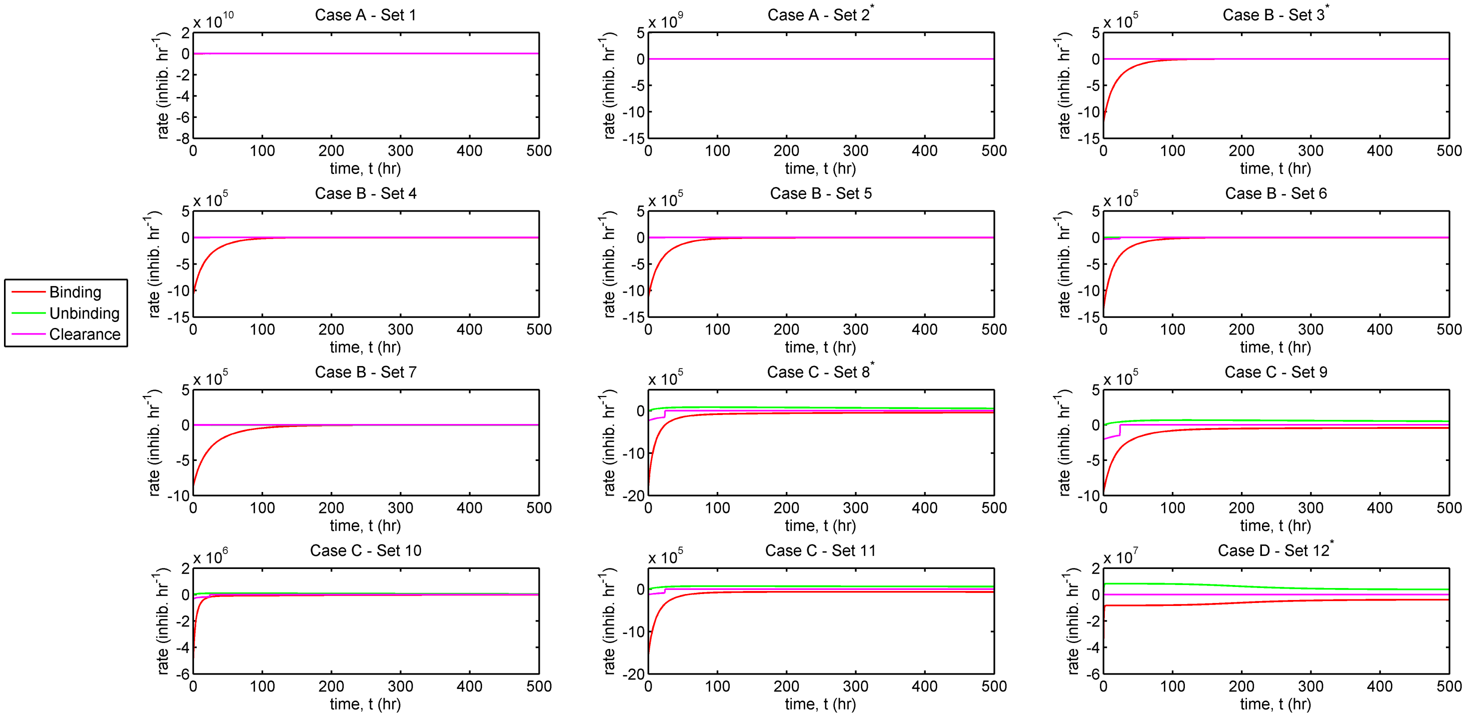

Figure K: Comparison of terms in the $\dot{A}_F$ equation (Eq 3) in the single inhibitor dose scenario. Simulations extend beyond the time span of the experiments, to 500 hours $\approx$ 21 days. The terms have settled to their steady-state values or are close to their steady-state values by t = 500 in all parameter sets. See Numerical solutions (main text) for discussion. Graphs marked with stars correspond to the parameter sets explored in the main text. Eqs 1–8 were solved using `ode15s`. See Tables 2 and A for parameter values.

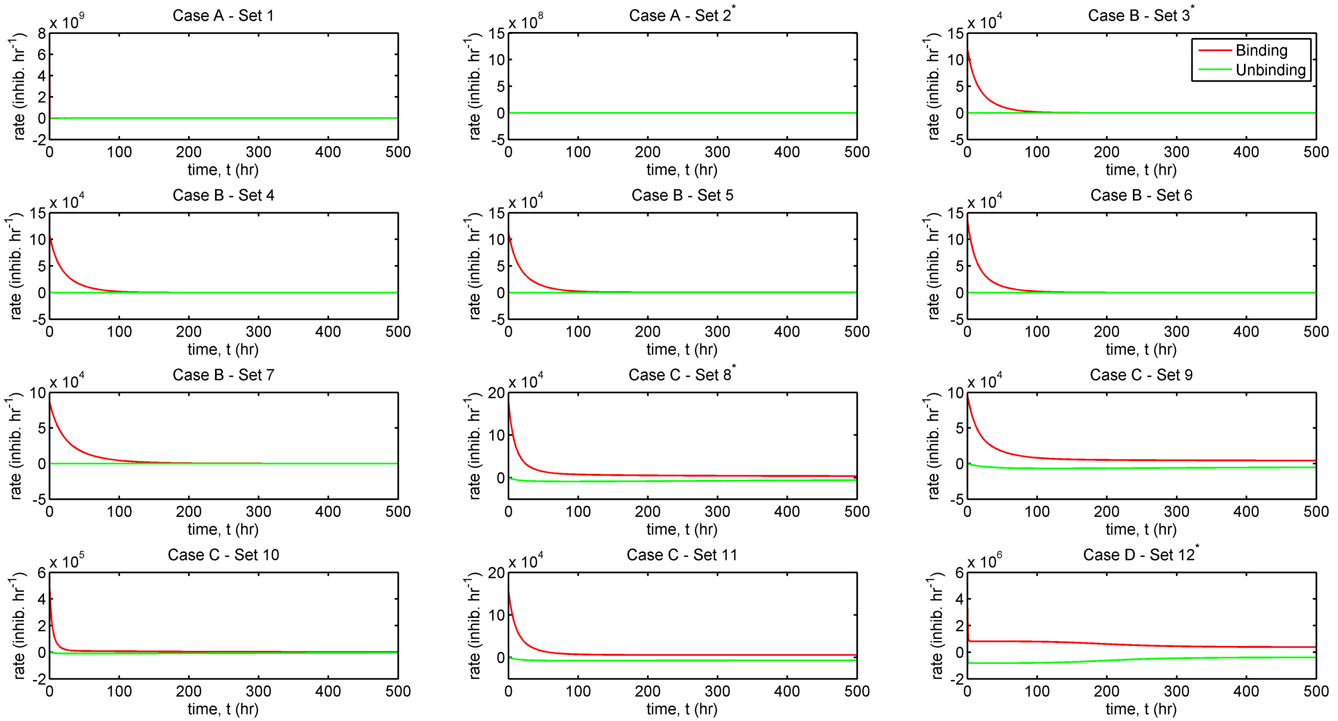

Figure L: Comparison of terms in the $\dot{A}_B$ equation (Eq 4) in the single inhibitor dose scenario. Simulations extend beyond the time span of the experiments, to 500 hours ≈ 21 days. The terms have settled to their steady-state values or are close to their steady-state values by $t = 500$ in all parameter sets. See Numerical solutions (main text) for discussion. Graphs marked with stars correspond to the parameter sets explored in the main text. Eqs 1–8 were solved using ode15s. See Tables 2 and A for parameter values.

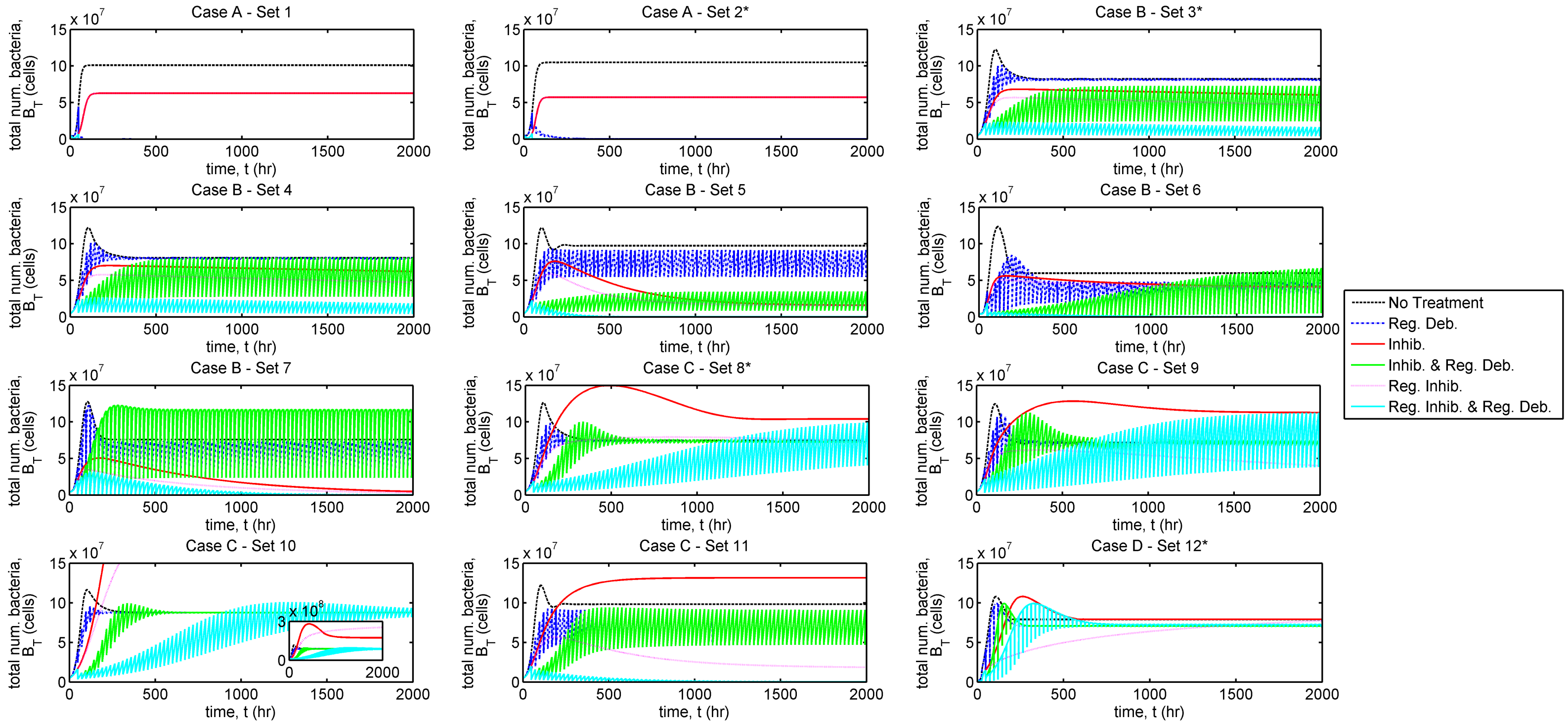

Figure M: Comparison of bacterial population dynamics under a variety of treatment regimes, including those which involve regular debridement and/or inhibitor dosing. The total number of bacteria, $B_T(t)$ $(= VB_F(t) + A_r B_B(t))$, is plotted in each case. Simulations extend beyond the time span of the experiments, to 2000 hours $\approx$ 83 days. The untreated and single inhibitor dose ('Inhib.') scenarios are identical to those in Fig D. See Treatment scenarios (main text) for a description of each treatment strategy. Case A: all treatments except single and regular inhibitor doses ('Reg. Inhib.') eradicate the bacterial population (such that $B_T(t) < 1$), where treatments combining inhibitor dosing with regular debridement are most effective; Case B: regular inhibitor dosing with regular debridement ('Reg. Inhib. and Reg. Deb.') is most effective, reducing the bacterial population size by an order of magnitude or more and almost eliminating it by $t = 2000$ hr in parameter set 5; Case C: all treatments are ineffective, except regular inhibitor dosing with regular debridement for parameter set 11, reducing the bacterial population size by several orders of magnitude; Case D: no treatment strategy is effective, all treatments resulting in long-term bacterial population sizes close to the untreated steady-state. Graphs marked with stars are those included in the main text. Eqs 1–8 were solved using ode15s. See Tables 2 and A for parameter values.

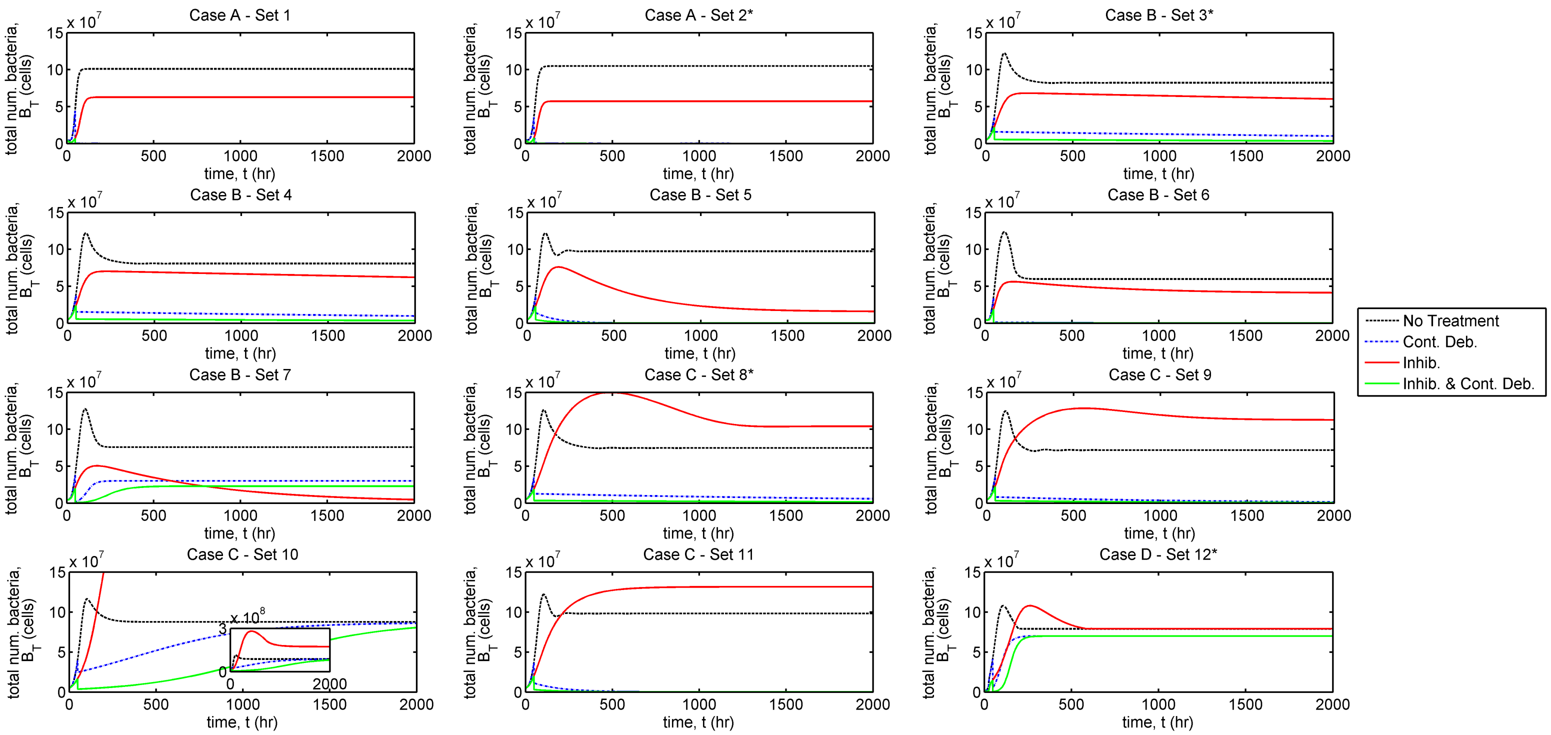

Figure N: Comparison of bacterial population dynamics under a variety of treatment regimes, including those which involve continuous debridement. The total number of bacteria, $B_T(t)$ $(= VB_F(t) + A_rB_B(t))$, is plotted in each case. Simulations extend beyond the time span of the experiments, to 2000 hours $\approx$ 83 days. The untreated and single inhibitor dose ('Inhib.') scenarios are identical to those in Fig D. Case A: all treatments except a single inhibitor dose ('Inhib.') eradicate the bacterial population (such that $B_T(t) < 1$); Case B: both continuous debridement ('Cont. Deb.') and inhibitor with continuous debridement ('Inhib. and Cont. Deb.') are effective, where inhibitor with continuous debridement is the most effective of the two. These treatments reduce the bacterial population size by an order of magnitude or more for all parameter sets, except Set 7 (where continuous debridement reduces the effectiveness of the inhibitor treatment), and eliminate (inhibitor with continuous debridement) or almost eliminate (continuous debridement) the bacterial population by $t = 2000$ hr for Set 5; Case C: only treatments involving continuous debridement are effective, reducing the bacterial population size by an order of magnitude or more, except for parameter set 10, where the population size approaches the untreated steady-state; Case D: no treatment strategy is effective. In all cases, inhibitor with continuous debridement is more effective than continuous debridement alone. Graphs marked with stars are those included in the main text. Eqs 1–8 were solved using `ode15s`. See Tables 2 and A for parameter values.

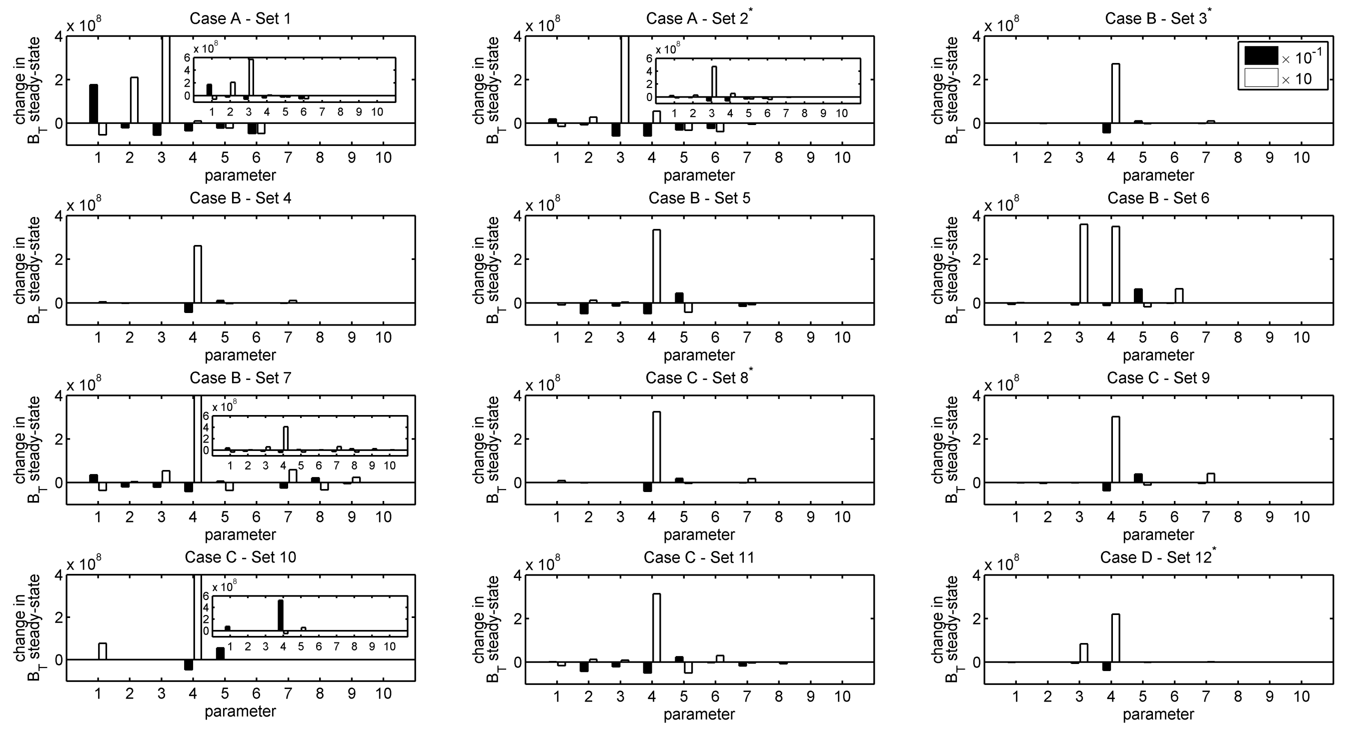

Figure O: Analysis to determine model sensitivity to each of the fitted parameters in the untreated scenario. Plots show the change in the steady-state value of the total number of bacteria, $B^*_{T_2}$ $(= VB^*_{F_2} + A_r B^*_{B_2})$, following a 10 fold increase or decrease in each parameter. Insets are given where the bar heights exceed the range of the axes. The model is most sensitive to the free and bound carrying capacities, K_F and K_B, and shows little sensitivity to the natural clearance rate of bacteria, $\tilde{\psi}_{Bac}$. Graphs marked with stars correspond to the parameter sets explored in the main text. Eqs 1–8 were solved using ode15s. See Tables 2 and A for the remaining parameter values. 1: r_F, 2: r_B, 3: K_F, 4: K_B, 5: α_{Bac}, 6: β_{Bac}, 7: δ_B, 8: η_{max}, 9: γ and 10: $\tilde{\psi}_{Bac}$.

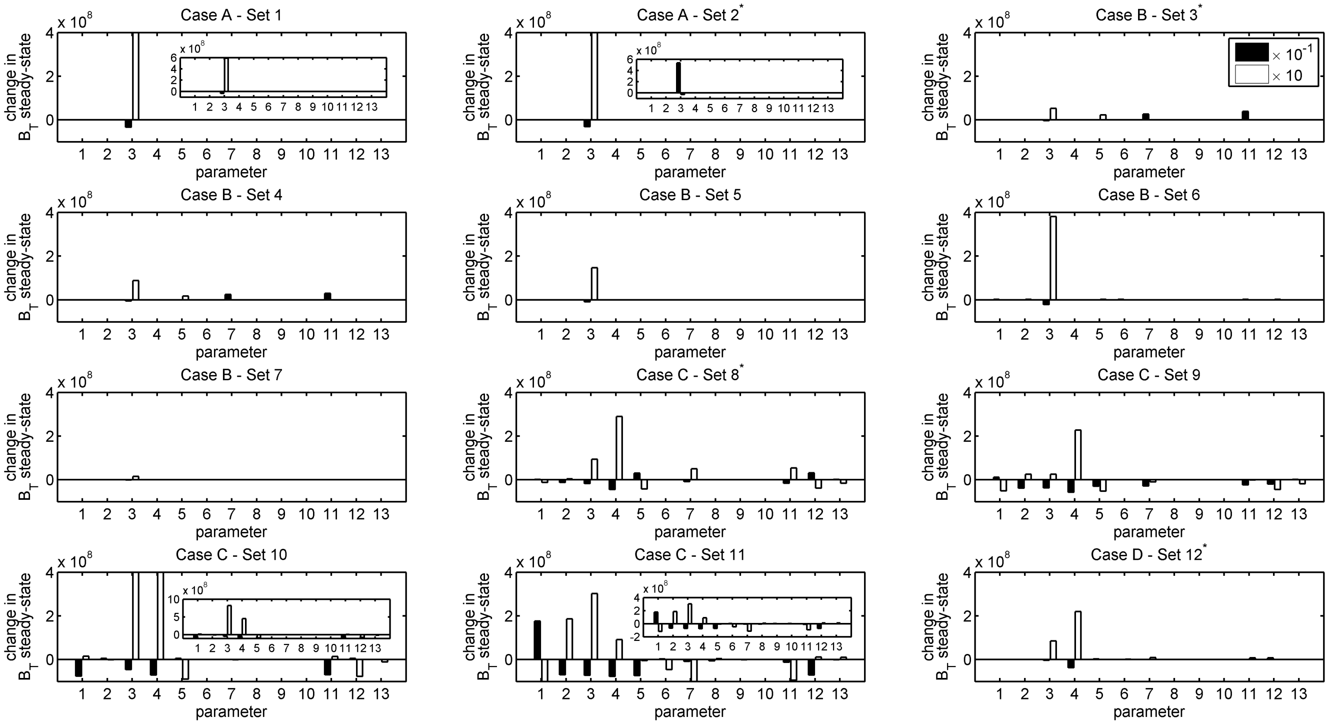

Figure P: Analysis to determine model sensitivity to each of the fitted parameters in the single inhibitor dose scenario. Plots show the change in the steady-state value of the total number of bacteria, $B^*_{T_2}$ $(= VB^*_{F_2} + A_r B^*_{B_2})$, following a 10 fold increase or decrease in each parameter. Insets are given where the bar heights exceed the range of the axes. The model is most sensitive to the free and bound carrying capacities, K_F and K_B, and shows little sensitivity to the bound daughter cell parameters, η_{max} and γ, and the natural clearance rate of bacteria, $\tilde{\psi}_{Bac}$. Graphs marked with stars correspond to the parameter sets explored in the main text. Eqs 1–8 were solved using ode15s. See Tables 2 and A for the remaining parameter values. 1: r_F, 2: r_B, 3: K_F, 4: K_B, 5: α_{Bac}, 6: β_{Bac}, 7: δ_B, 8: η_{max}, 9: γ, 10: $\tilde{\psi}_{Bac}$, 11: α_A, 12: β_A and 13: $\tilde{\psi}_A$.

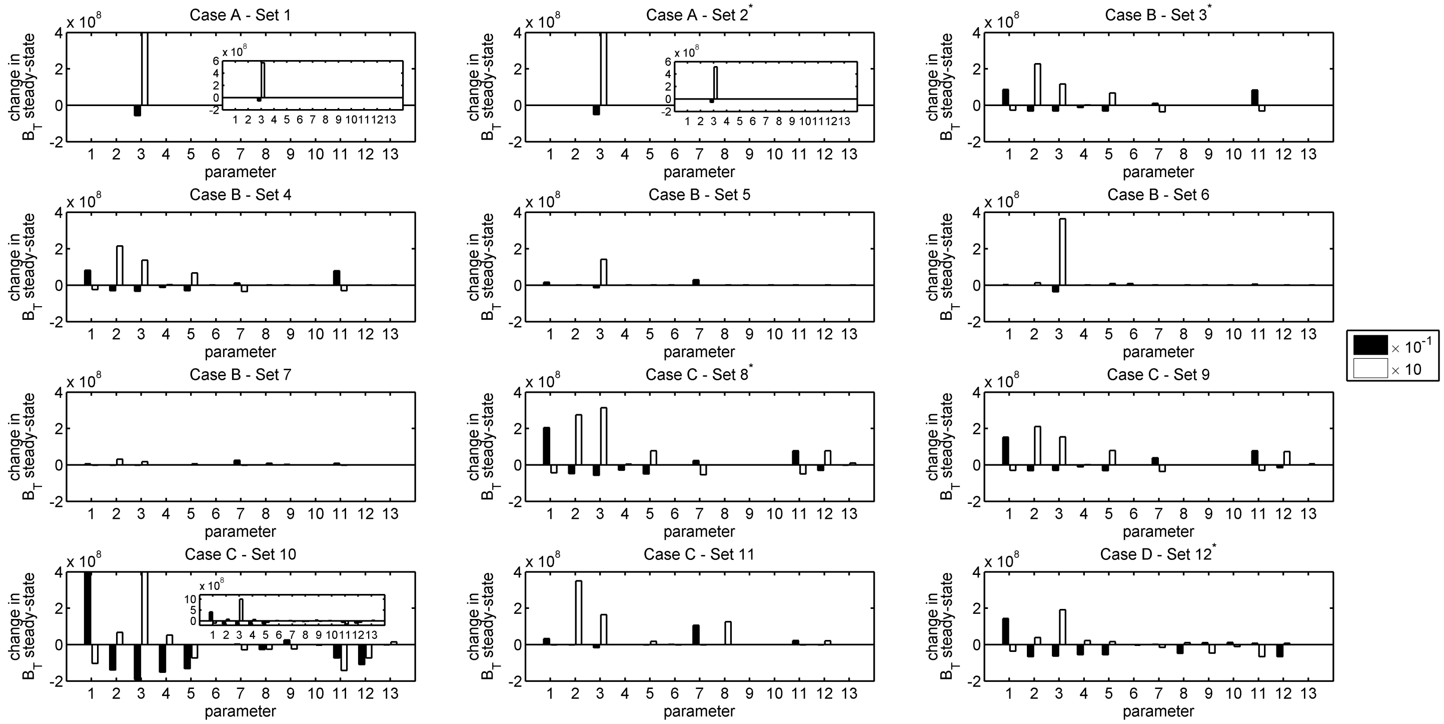

Figure Q: Analysis to determine model sensitivity to each of the fitted parameters in the regular inhibitor dosing scenario. Plots show the change in the steady-state value of the total number of bacteria, $B^*_{T_2}$ $(= VB^*_{F_2} + A_r B^*_{B_2})$, following a 10 fold increase or decrease in each parameter. Insets are given where the bar heights exceed the range of the axes. The model is most sensitive to the free and bound intrinsic growth rates, r_F and r_B, and the free carrying capacity, K_F, and shows little sensitivity to the rate of bacterial unbinding, β_{Bac}, and the natural clearance rates of bacteria and inhibitors, $\tilde{\psi}_{Bac}$ and $\tilde{\psi}_A$. Graphs marked with stars correspond to the parameter sets explored in the main text. Eqs 1–8 were solved using `ode15s`. See Tables 2 and A for the remaining parameter values. 1: r_F, 2: r_B, 3: K_F, 4: K_B, 5: α_{Bac}, 6: β_{Bac}, 7: δ_B, 8: η_{max}, 9: γ, 10: $\tilde{\psi}_{Bac}$, 11: α_A, 12: β_A and 13: $\tilde{\psi}_A$.

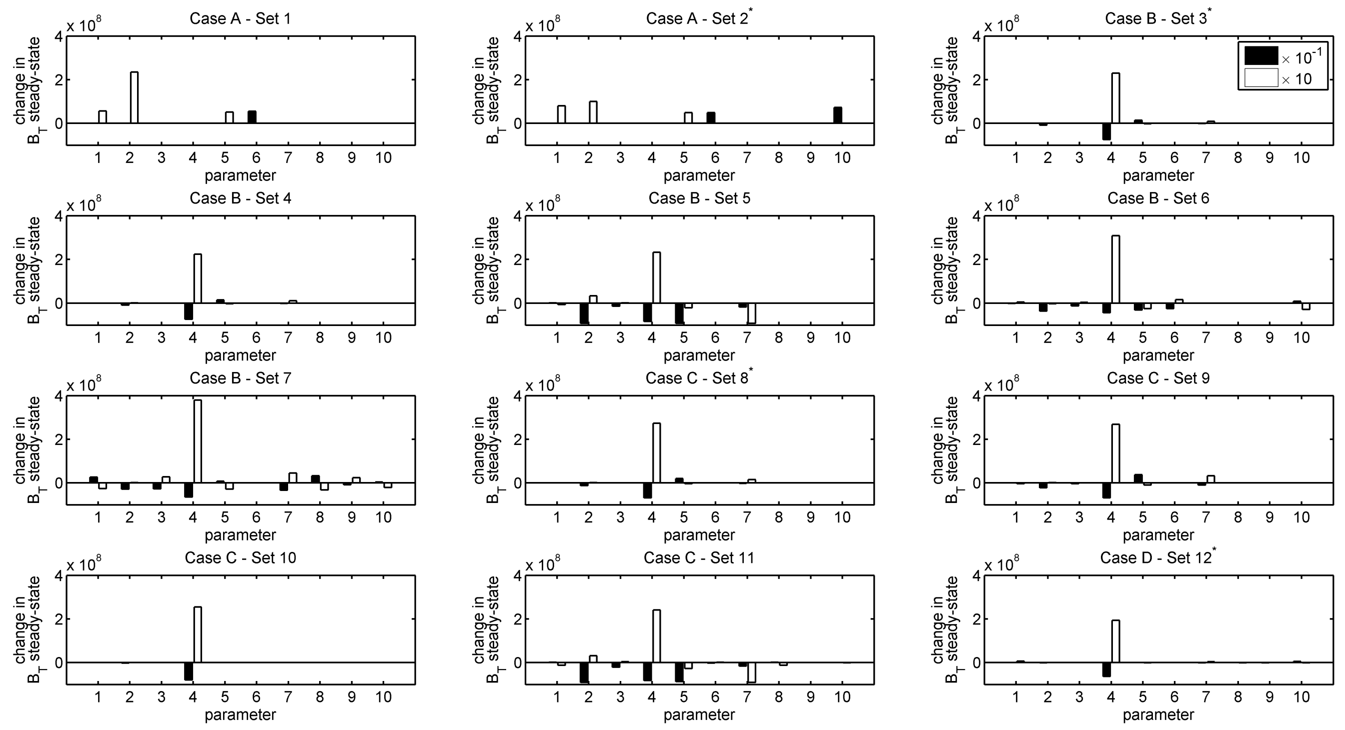

Figure R: Analysis to determine model sensitivity to each of the fitted parameters in the regular debridement scenario. Plots show the change in the steady-state value of the total number of bacteria, $B^*_{T_2}$ $(= VB^*_{F_2} + A_r B^*_{B_2})$, following a 10 fold increase or decrease in each parameter. The model is most sensitive to the bound carrying capacity, K_B, and shows sensitivity to all parameters. Graphs marked with stars correspond to the parameter sets explored in the main text. Eqs 1–8 were solved using `ode15s`. See Tables 2 and A for the remaining parameter values. 1: r_F, 2: r_B, 3: K_F, 4: K_B, 5: α_{Bac}, 6: β_{Bac}, 7: δ_B, 8: η_{max}, 9: γ and 10: $\tilde{\psi}_{Bac}$.

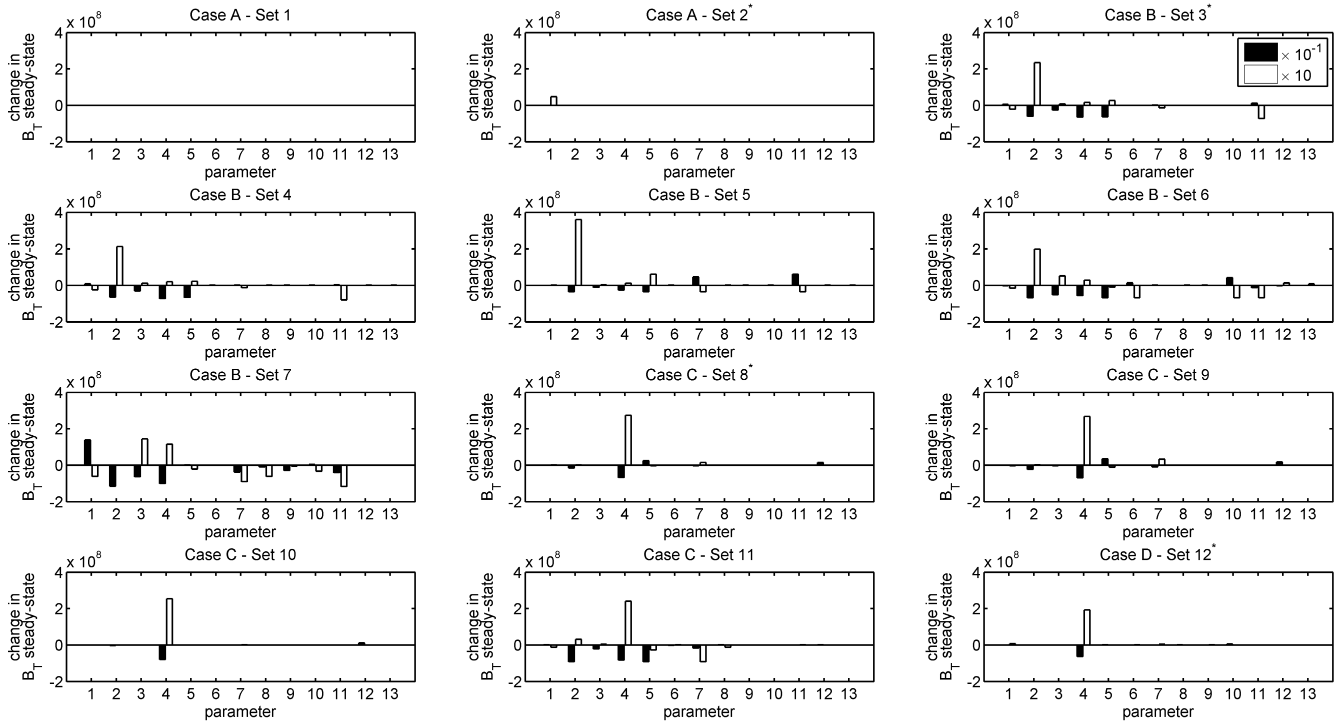

Figure S: Analysis to determine model sensitivity to each of the fitted parameters in the single inhibitor dose with regular debridement scenario. Plots show the change in the steady-state value of the total number of bacteria, $B^*_{T_2}$ $(= VB^*_{F_2} + A_r B^*_{B_2})$, following a 10 fold increase or decrease in each parameter. The model is most sensitive to the bound intrinsic growth rate, r_B, and the bound carrying capacity, K_B, and shows little sensitivity to the bound daughter cell parameter γ, the inhibitor unbinding rate, β_A, and the natural clearance rate of inhibitors, $\tilde{\psi}_A$. Graphs marked with stars correspond to the parameter sets explored in the main text. Eqs 1–8 were solved using ode15s. See Tables 2 and A for the remaining parameter values. 1: r_F, 2: r_B, 3: K_F, 4: K_B, 5: α_{Bac}, 6: β_{Bac}, 7: δ_B, 8: η_{max}, 9: γ, 10: $\tilde{\psi}_{Bac}$, 11: α_A, 12: β_A and 13: $\tilde{\psi}_A$.

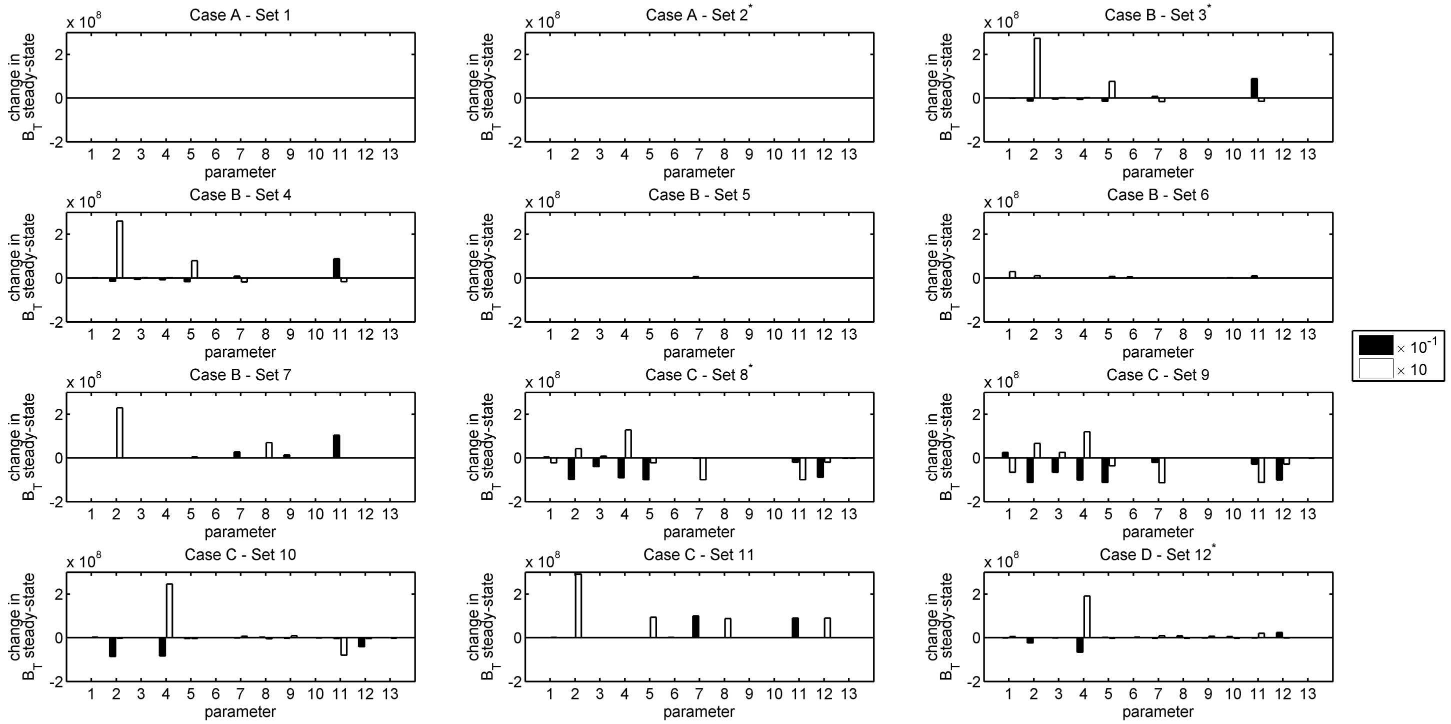

Figure T: Analysis to determine model sensitivity to each of the fitted parameters in the regular inhibitor dosing with regular debridement scenario. Plots show the change in the steady-state value of the total number of bacteria, $B^*_{T_2}$ $(= VB^*_{F_2} + A_r B^*_{B_2})$, following a 10 fold increase or decrease in each parameter. The model is most sensitive to the bound intrinsic growth rate, r_B, and the bound carrying capacity, K_B, and shows little sensitivity to the rate of bacterial unbinding, β_{Bac}, the bound daughter cell parameter, γ, and the natural clearance rates of bacteria and inhibitors, $\tilde{\psi}_{Bac}$ and $\tilde{\psi}_A$. Graphs marked with stars correspond to the parameter sets explored in the main text. Eqs 1–8 were solved using ode15s. See Tables 2 and A for the remaining parameter values. 1: r_F, 2: r_B, 3: K_F, 4: K_B, 5: α_{Bac}, 6: β_{Bac}, 7: δ_B, 8: η_{max}, 9: γ, 10: $\tilde{\psi}_{Bac}$, 11: α_A, 12: β_A and 13: $\tilde{\psi}_A$.

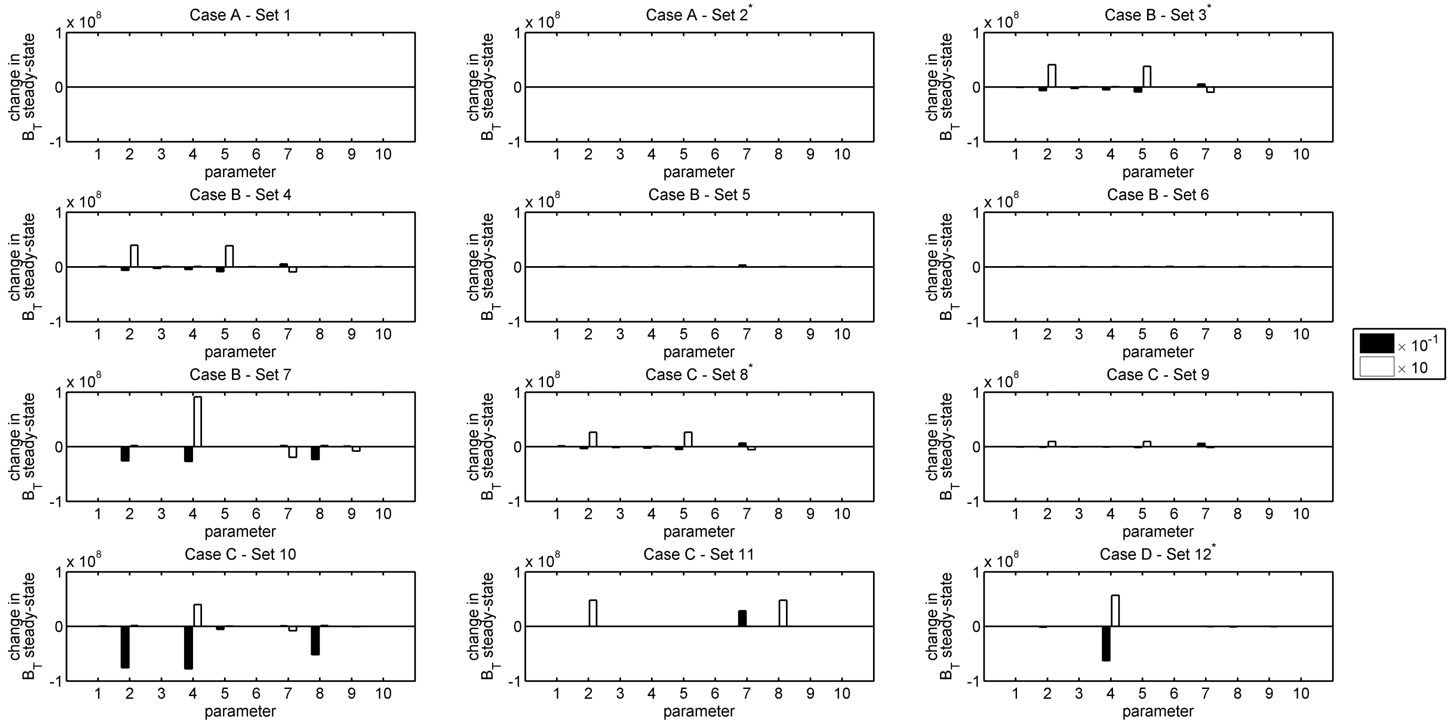

Figure U: Analysis to determine model sensitivity to each of the fitted parameters in the continuous debridement scenario. Plots show the change in the steady-state value of the total number of bacteria, $B^*_{T_2}$ $(= VB^*_{F_2} + A_rB^*_{B_2})$, following a 10 fold increase or decrease in each parameter. The model is most sensitive to the bound intrinsic growth rate, r_B, the bound carrying capacity, K_B, and the bacterial binding rate, α_{Bac}, and shows little sensitivity to the free intrinsic growth rate, r_F, the free carrying capacity, K_F, the bacterial unbinding rate, β_{Bac}, the bound daughter cell parameter γ, and the natural clearance rate of bacteria, $\tilde{\psi}_{Bac}$. Graphs marked with stars correspond to the parameter sets explored in the main text. Eqs 1–8 were solved using ode15s. See Tables 2 and A for the remaining parameter values. 1: r_F, 2: r_B, 3: K_F, 4: K_B, 5: α_{Bac}, 6: β_{Bac}, 7: δ_B, 8: η_{max}, 9: γ and 10: $\tilde{\psi}_{Bac}$.

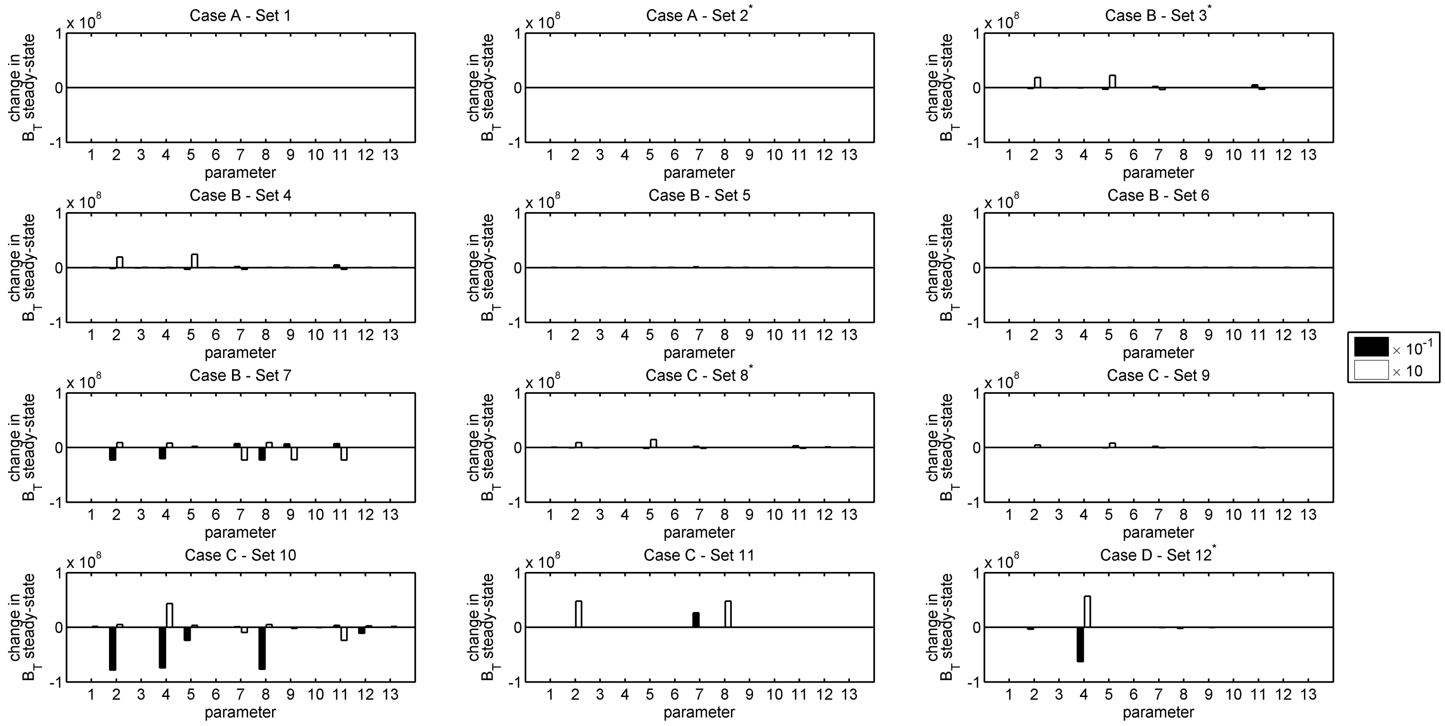

Figure V: Analysis to determine model sensitivity to each of the fitted parameters in the single inhibitor dose with continuous debridement scenario. Plots show the change in the steady-state value of the total number of bacteria, $B^*_{T_2}$ $(= VB^*_{F_2} + A_r B^*_{B_2})$, following a 10 fold increase or decrease in each parameter. The model is most sensitive to the bound intrinsic growth rate, r_B, the bound carrying capacity, K_B, the bacterial binding rate, α_{Bac}, the phagocytosis rate, δ_B, and the bound daughter cell parameter, η_{max}, and shows little sensitivity to the free intrinsic growth rate, r_F, the free carrying capacity, K_F, the rate of bacterial unbinding, β_{Bac}, and the natural clearance rates of bacteria and inhibitors, $\tilde{\psi}_{Bac}$ and $\tilde{\psi}_A$. Graphs marked with stars correspond to the parameter sets explored in the main text. Eqs 1–8 were solved using `ode15s`. See Tables 2 and A for the remaining parameter values. 1: r_F, 2: r_B, 3: K_F, 4: K_B, 5: α_{Bac}, 6: β_{Bac}, 7: δ_B, 8: η_{max}, 9: γ, 10: $\tilde{\psi}_{Bac}$, 11: α_A, 12: β_A and 13: $\tilde{\psi}_A$.

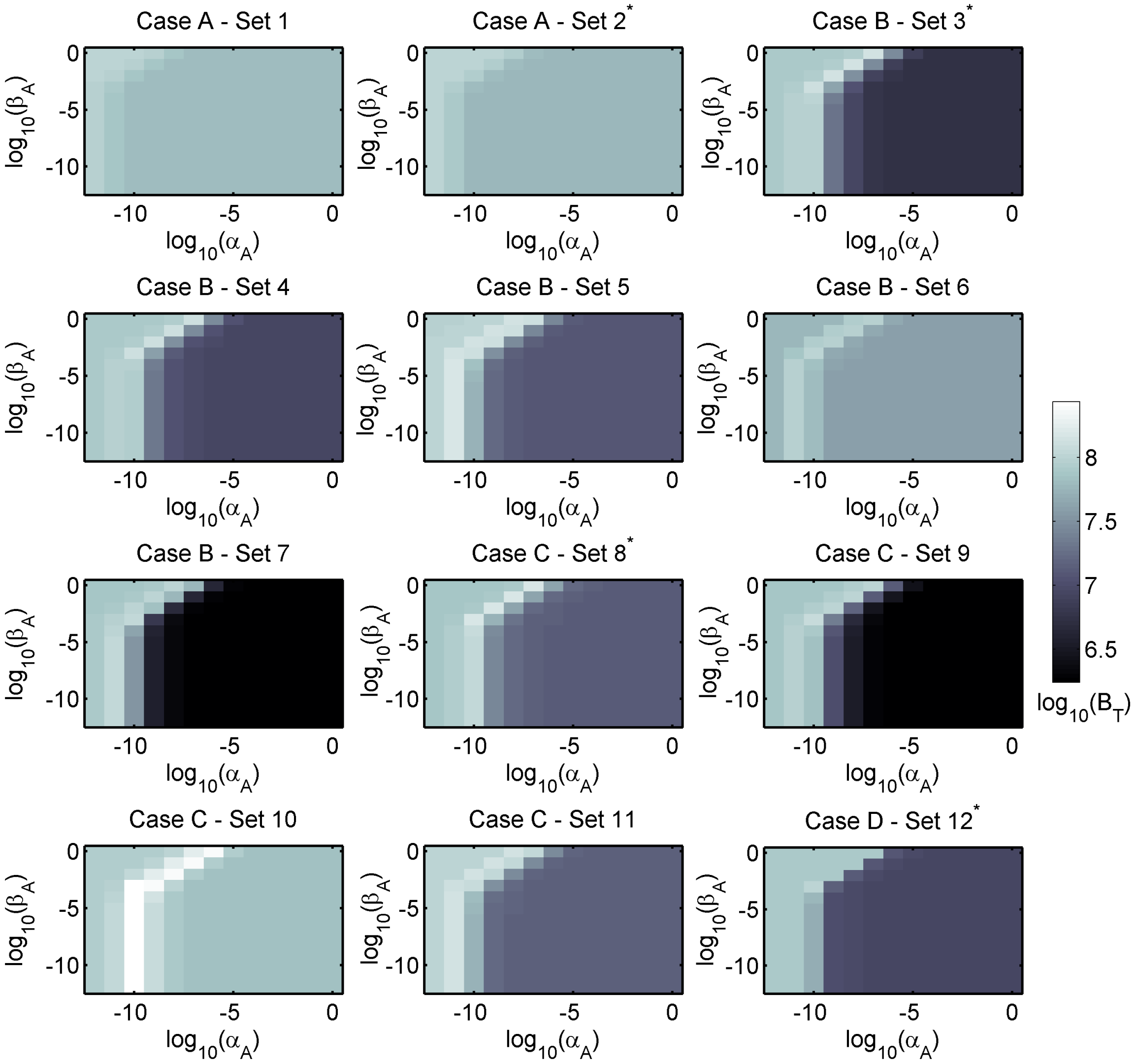

Figure W: Sensitivity analysis to determine the effect of varying inhibitor binding (α_A) and unbinding (β_A) rates upon the efficacy of a single inhibitor dose, where $A_{F_{init}} = 6.12 \times 10^7$ inhibitors cm^{-3} (the standard value). For each panel, α_A and $\beta_A = 10^{-12}$, 10^{-11}, ..., 10^{-1} and 1, where a $\log_{10}$ scale is used on both axes. The value of $\log_{10}(B_T(672))$ is plotted at each point in parameter space, where $B_T(672)$ $(= VB_F(672) + A_r B_B(672))$ is the total number of bacteria after 4 weeks (672 hr). In all cases treatment fails to eliminate the bacterial population (such that $B_T(672) < 1$), though the number of bacteria is generally reduced with increasing α_A and decreasing β_A. Graphs marked with stars correspond to the parameter sets explored in the main text. Eqs 1–8 were solved using ode15s. See Tables 2 and A for the remaining parameter values.

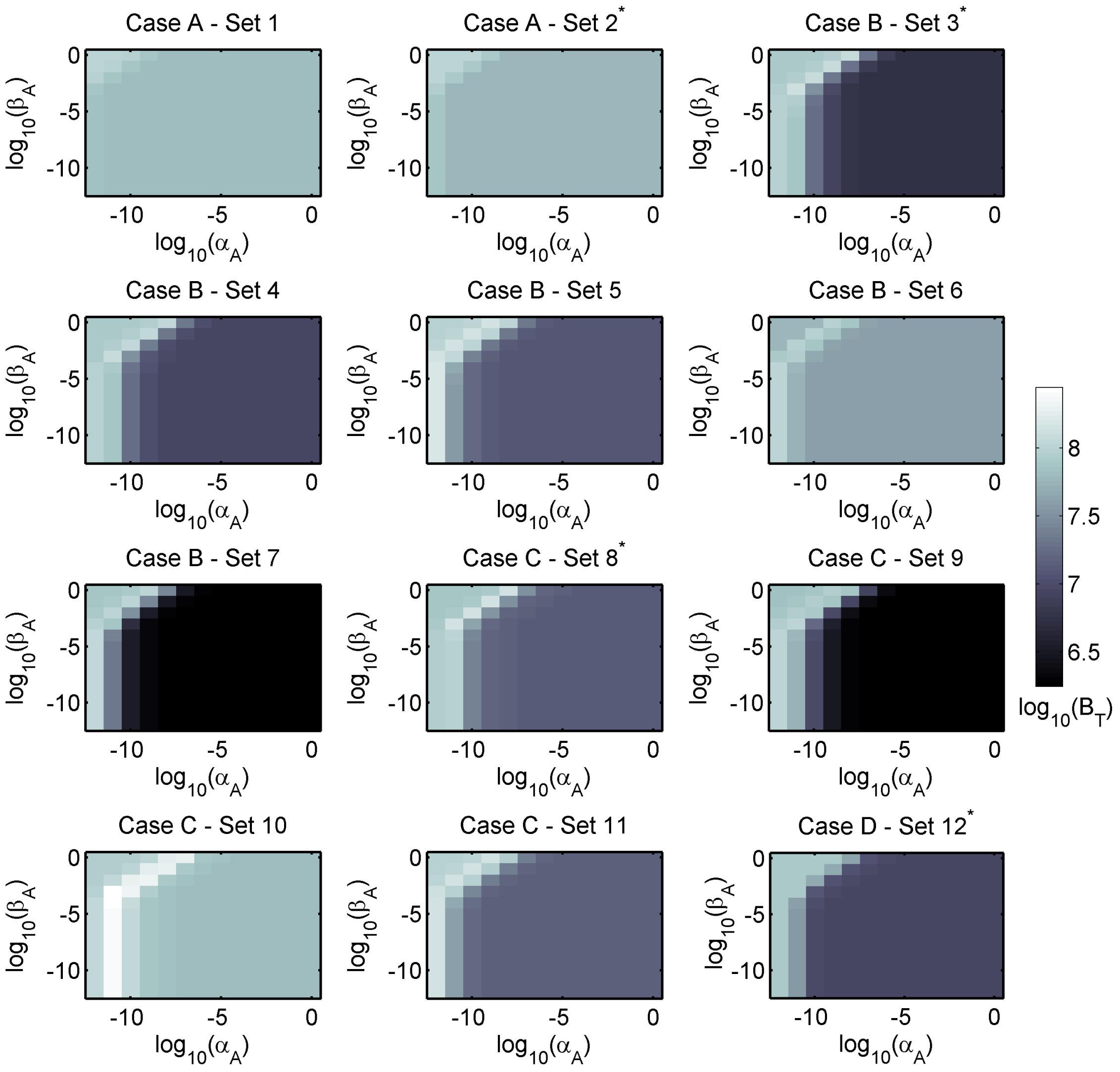

Figure X: Sensitivity analysis to determine the effect of varying inhibitor binding (α_A) and unbinding (β_A) rates upon the efficacy of a single inhibitor dose, where $A_{F_{init}} = 6.12 \times 10^8$ inhibitors cm^{-3} (10× the standard value). For each panel, α_A and $\beta_A = 10^{-12}, 10^{-11}, \ldots, 10^{-1}$ and 1, where a $\log_{10}$ scale is used on both axes. The value of $\log_{10}(B_T(672))$ is plotted at each point in parameter space, where $B_T(672)$ $(= VB_F(672) + A_r B_B(672))$ is the total number of bacteria after 4 weeks (672 hr). In all cases treatment fails to eliminate the bacterial population (such that $B_T(672) < 1$), though the number of bacteria is generally reduced with increasing α_A and decreasing β_A. Treatment is slightly more effective than when the standard number of inhibitors is used (compare Fig W). Graphs marked with stars correspond to the parameter sets explored in the main text. Eqs 1–8 were solved using `ode15s`. See Tables 2 and A for the remaining parameter values.

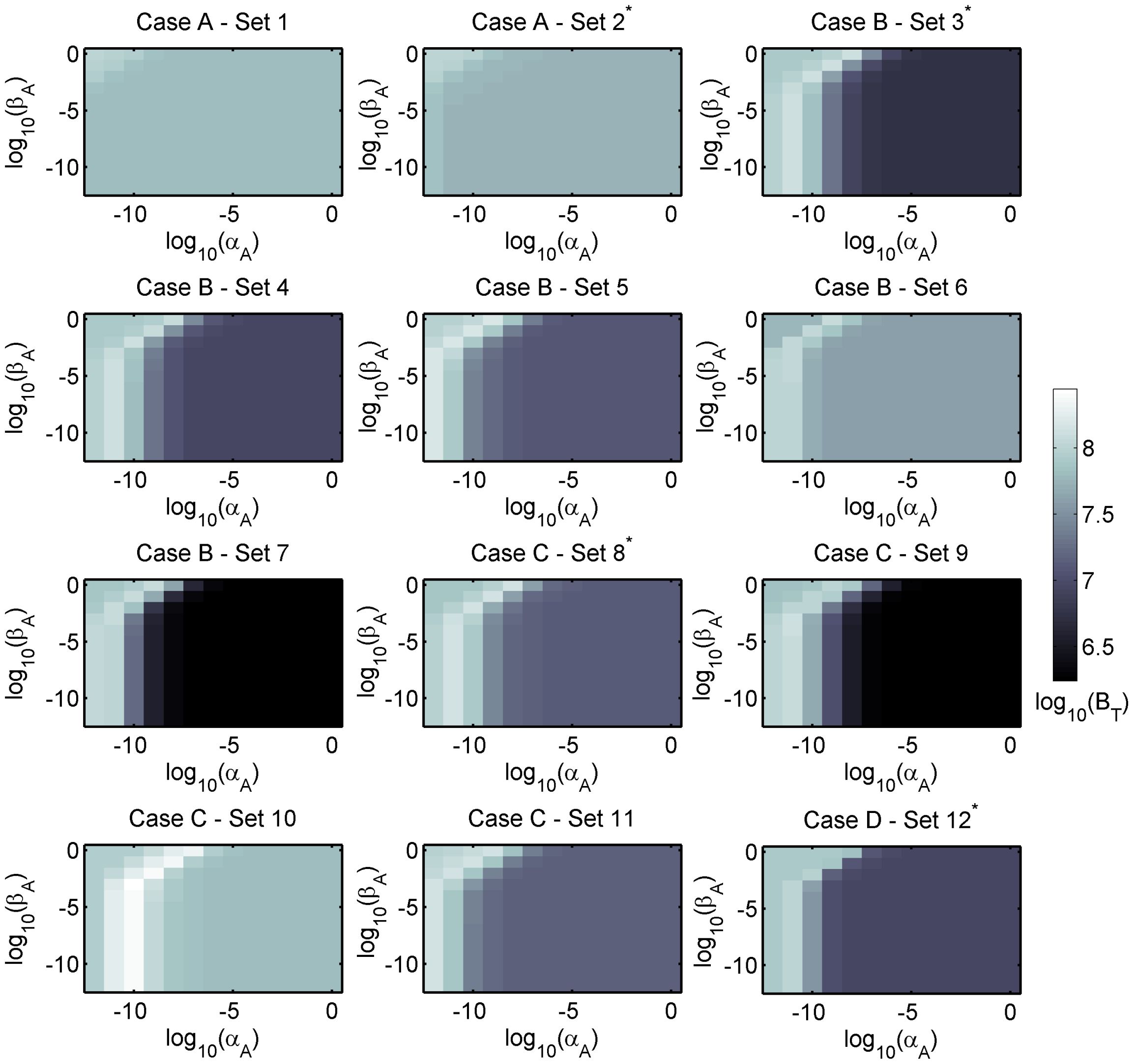

Figure Y: Sensitivity analysis to determine the effect of varying inhibitor binding (α_A) and unbinding (β_A) rates upon the efficacy of repeated inhibitor doses, where the initial ($A_{F_{init}}$) and subsequent doses each equal 6.12×10^7 inhibitors cm^{-3} (the standard value). For each panel, α_A and $\beta_A = 10^{-12}, 10^{-11}, \ldots, 10^{-1}$ and 1, where a $\log_{10}$ scale is used on both axes. The value of $\log_{10}(B_T(672))$ is plotted at each point in parameter space, where $B_T(672)$ $(= VB_F(672) + A_r B_B(672))$ is the total number of bacteria after 4 weeks (672 hr). In all cases treatment fails to eliminate the bacterial population (such that $B_T(672) < 1$), though the number of bacteria is generally reduced with increasing α_A and decreasing β_A. Treatment efficacy is similar to the single inhibitor dose scenario (compare Fig W). Graphs marked with stars correspond to the parameter sets explored in the main text. Eqs 1–8 were solved using ode15s. See Tables 2 and A for the remaining parameter values.

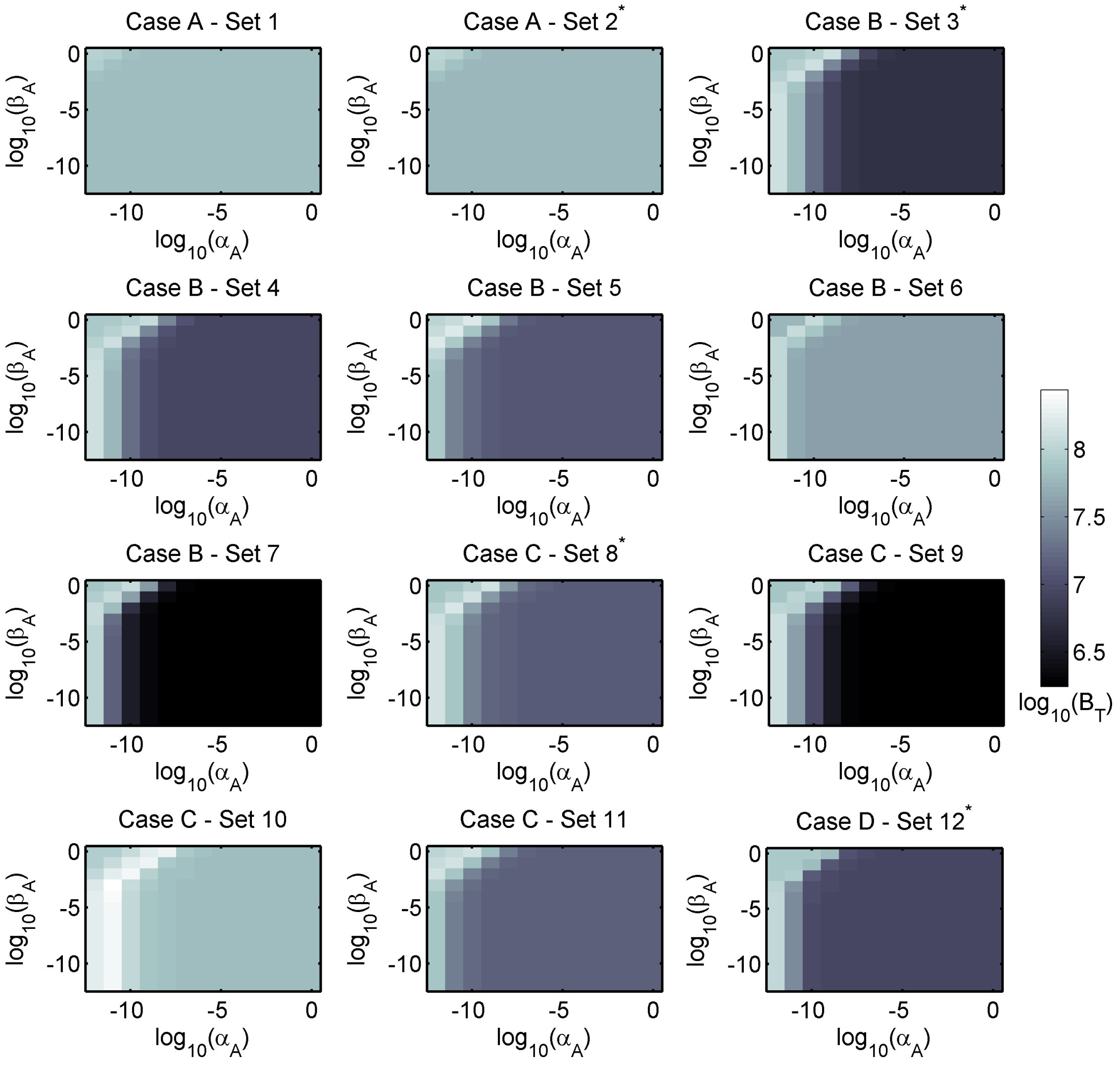

Figure Z: Sensitivity analysis to determine the effect of varying inhibitor binding (α_A) and unbinding (β_A) rates upon the efficacy of repeated inhibitor doses, where the initial ($A_{F_{init}}$) and subsequent doses each equal 6.12×10^8 inhibitors cm^{-3} (10× the standard value). For each panel, α_A and β_A = 10^{-12}, 10^{-11}, ..., 10^{-1} and 1, where a $\log_{10}$ scale is used on both axes. The value of $\log_{10}(B_T(672))$ is plotted at each point in parameter space, where $B_T(672)$ (= $VB_F(672) + A_r B_B(672)$) is the total number of bacteria after 4 weeks (672 hr). In all cases treatment fails to eliminate the bacterial population (such that $B_T(672) < 1$), though the number of bacteria is generally reduced with increasing α_A and decreasing β_A. Treatment is slightly more effective than when the standard number of inhibitors is used (compare Fig Y) and efficacy is similar to the single inhibitor dose scenario when 10× the standard number of inhibitors is used (compare Fig X). Graphs marked with stars correspond to the parameter sets explored in the main text. Eqs 1–8 were solved using `ode15s`. See Tables 2 and A for the remaining parameter values.

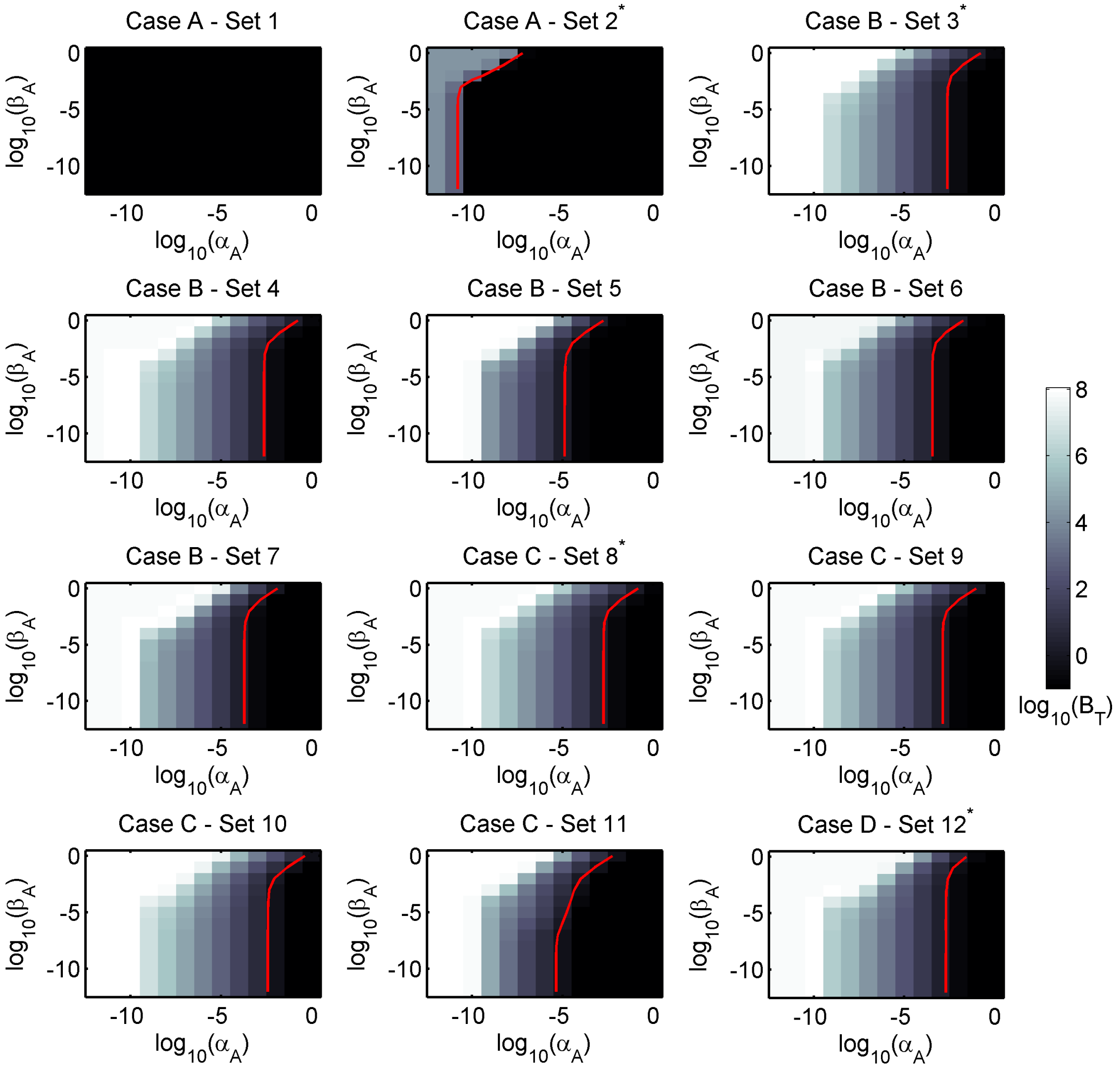

Figure AA: Sensitivity analysis to determine the effect of varying inhibitor binding (α_A) and unbinding (β_A) rates upon the efficacy of a single inhibitor dose combined with regular debridement, where $A_{F_{init}} = 6.12 \times 10^7$ inhibitors cm^{-3} (the standard value). For each panel, α_A and $\beta_A = 10^{-12}, 10^{-11}, \ldots, 10^{-1}$ and 1, where a $\log_{10}$ scale is used on both axes. The value of $\log_{10}(B_T(672))$ is plotted at each point in parameter space, where $B_T(672)$ $(= VB_F(672) + A_r B_B(672))$ is the total number of bacteria after 4 weeks (672 hr). The colour scheme is calibrated to maximise clarity, such that values of $\log_{10}(B_T(672)) \leq -1$ appear in black. The red curve (colour online) traces the contour along which $B_T(672) = 1$, such that $B_T(672) > 1$ to the left and $B_T(672) < 1$ to the right of this curve. In Set 1 all treatments eliminate the bacterial population (such that $B_T(672) < 1$), while, for the remaining parameter sets, treatment is effective for sufficiently high α_A and low β_A. Treatment is much more effective than either single or repeated inhibitor doses (compare Figs W–Z). Graphs marked with stars correspond to the parameter sets explored in the main text. Eqs 1–8 were solved using `ode15s`. See Tables 2 and A for the remaining parameter values.

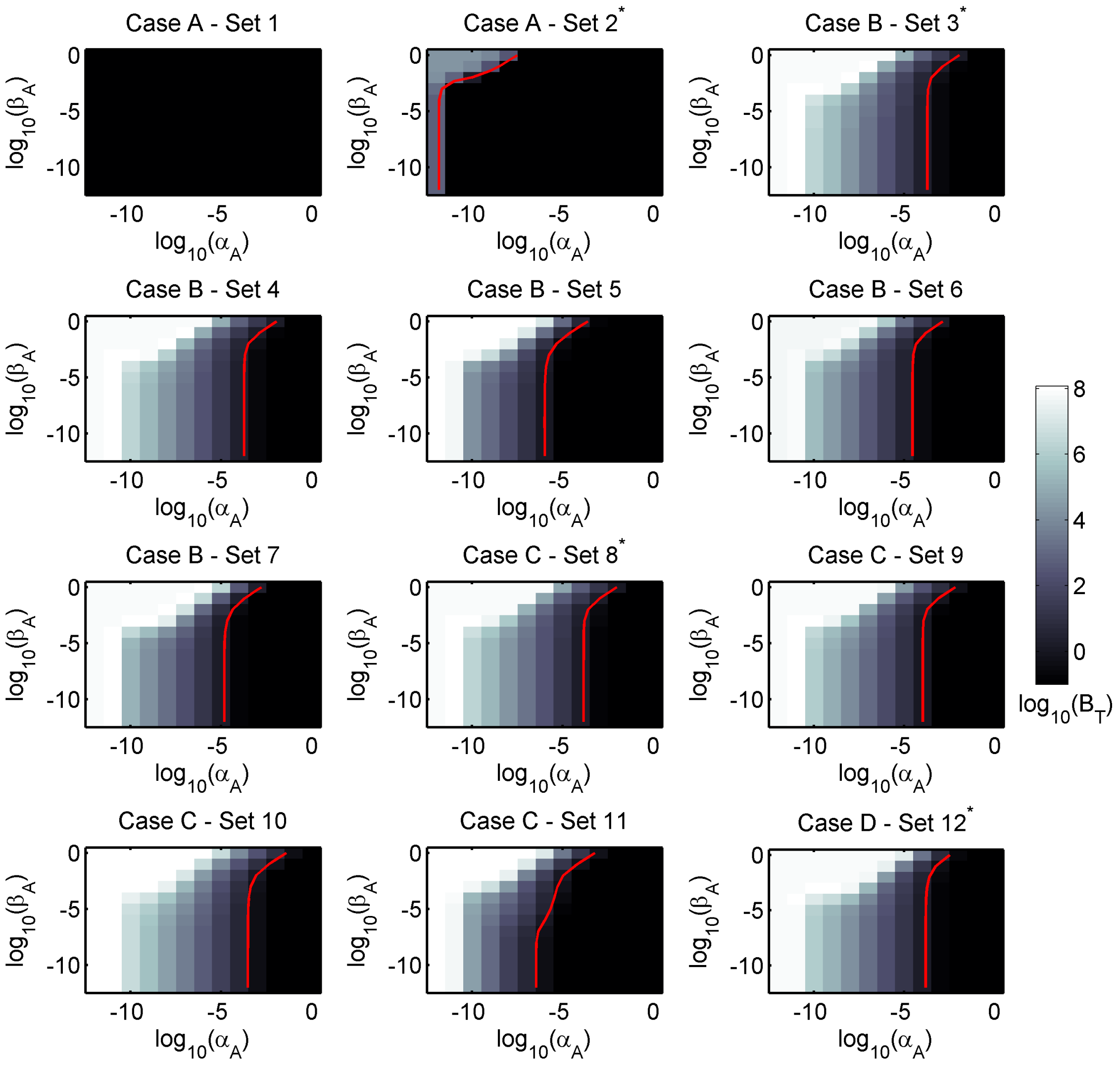

Figure AB: Sensitivity analysis to determine the effect of varying inhibitor binding (α_A) and unbinding (β_A) rates upon the efficacy of a single inhibitor dose combined with regular debridement, where $A_{F_{init}} = 6.12 \times 10^8$ inhibitors cm^{-3} (10× the standard value). For each panel, α_A and $\beta_A = 10^{-12}$, 10^{-11}, ..., 10^{-1} and 1, where a $\log_{10}$ scale is used on both axes. The value of $\log_{10}(B_T(672))$ is plotted at each point in parameter space, where $B_T(672)$ (= $VB_F(672) + A_r B_B(672)$) is the total number of bacteria after 4 weeks (672 hr). The colour scheme is calibrated to maximise clarity, such that values of $\log_{10}(B_T(672)) \leq -1$ appear in black. The red curve (colour online) traces the contour along which $B_T(672) = 1$, such that $B_T(672) > 1$ to the left and $B_T(672) < 1$ to the right of this curve. In Set 1 all treatments eliminate the bacterial population (such that $B_T(672) < 1$), while, for the remaining parameter sets, treatment is effective for sufficiently high α_A and low β_A. Treatment is more effective than when the standard number of inhibitors is used (compare Fig AA). Graphs marked with stars correspond to the parameter sets explored in the main text. Eqs 1–8 were solved using ode15s. See Tables 2 and A for the remaining parameter values.

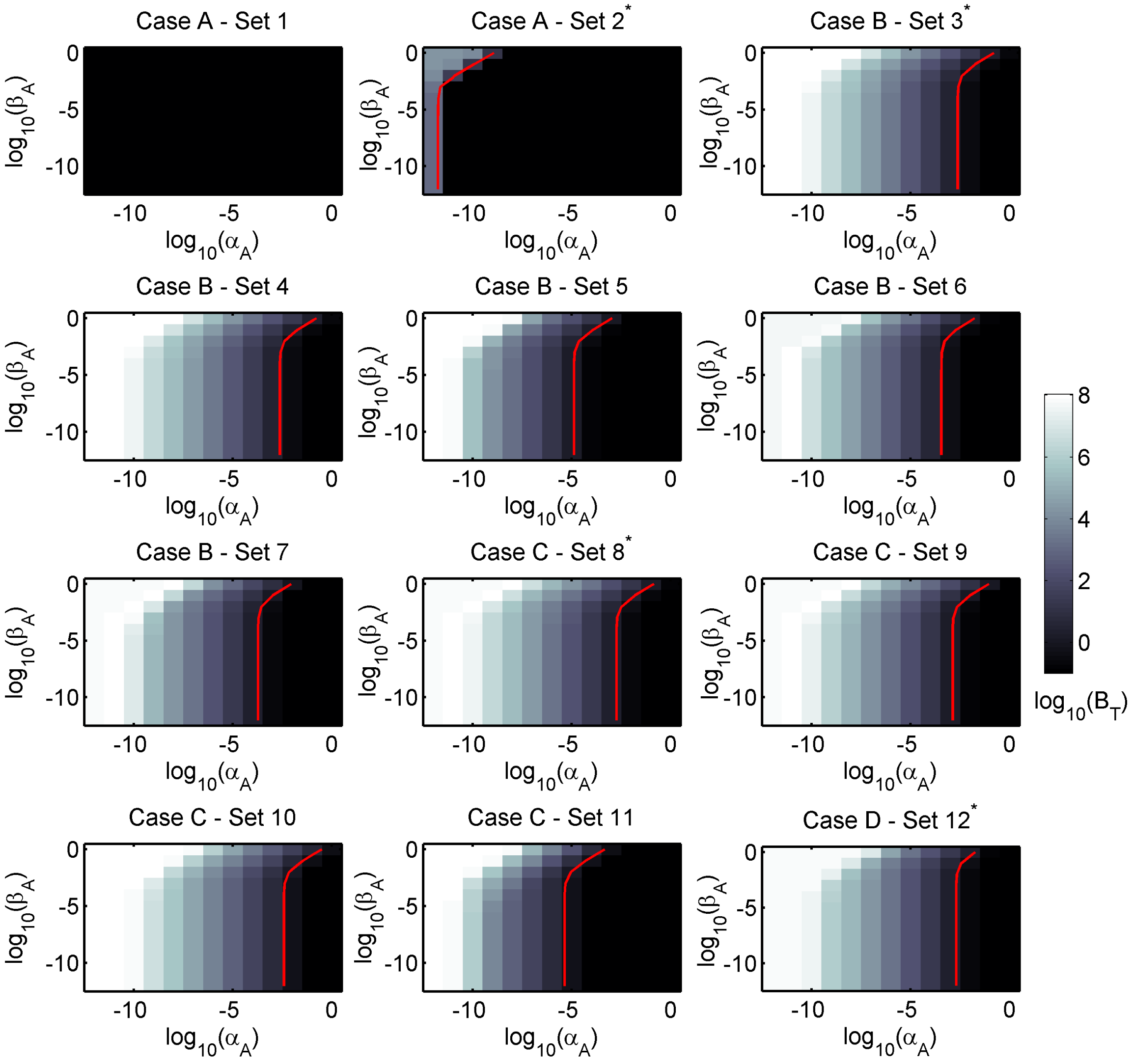

Figure AC: Sensitivity analysis to determine the effect of varying inhibitor binding (α_A) and unbinding (β_A) rates upon the efficacy of regular inhibitor doses combined with regular debridement, where the initial ($A_{F_{init}}$) and subsequent inhibitor doses each equal 6.12×10^7 inhibitors cm^{-3} (the standard value). For each panel, α_A and β_A = 10^{-12}, 10^{-11}, ..., 10^{-1} and 1, where a $\log_{10}$ scale is used on both axes. The value of $\log_{10}(B_T(672))$ is plotted at each point in parameter space, where $B_T(672)$ (= $VB_F(672) + A_r B_B(672)$) is the total number of bacteria after 4 weeks (672 hr). The colour scheme is calibrated to maximise clarity, such that values of $\log_{10}(B_T(672)) \leq -1$ appear in black. The red curve (colour online) traces the contour along which $B_T(672) = 1$, such that $B_T(672) > 1$ to the left and $B_T(672) < 1$ to the right of this curve. In Set 1 all treatments eliminate the bacterial population (such that $B_T(672) < 1$), while, for the remaining parameter sets, treatment is effective for sufficiently high α_A and low β_A. Treatment is slightly more effective than treatments combining a single inhibitor dose with regular debridement (compare Fig AA). Graphs marked with stars correspond to the parameter sets explored in the main text. Eqs 1–8 were solved using ode15s. See Tables 2 and A for the remaining parameter values.

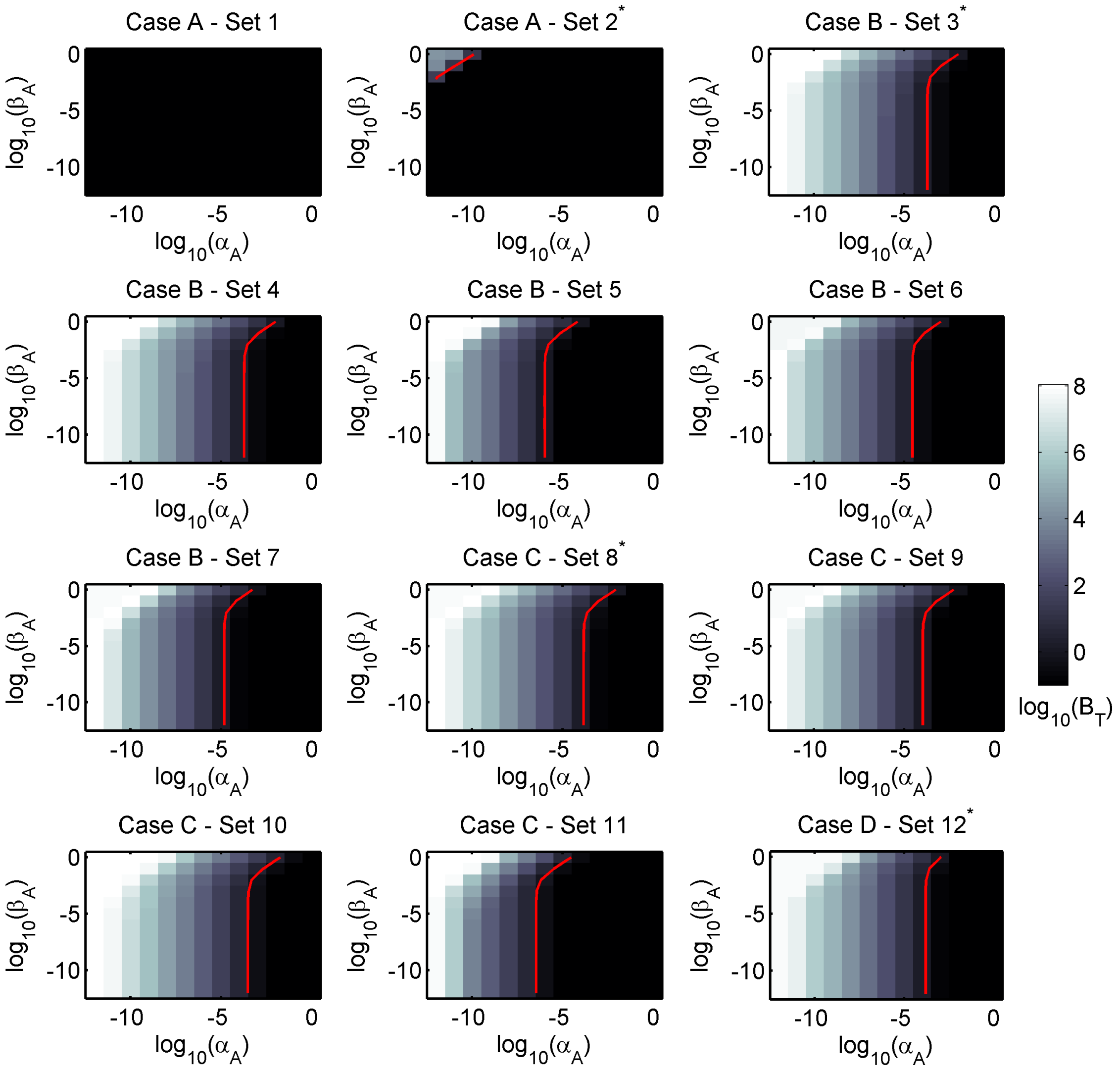

Figure AD: Sensitivity analysis to determine the effect of varying inhibitor binding (α_A) and unbinding (β_A) rates upon the efficacy of regular inhibitor doses combined with regular debridement, where the initial ($A_{F_{init}}$) and subsequent inhibitor doses each equal 6.12×10^8 inhibitors cm^{-3} (10× the standard value). For each panel, α_A and β_A = 10^{-12}, 10^{-11}, ..., 10^{-1} and 1, where a $\log_{10}$ scale is used on both axes. The value of $\log_{10}(B_T(672))$ is plotted at each point in parameter space, where $B_T(672)$ (= $VB_F(672) + A_r B_B(672)$) is the total number of bacteria after 4 weeks (672 hr). The colour scheme is calibrated to maximise clarity, such that values of $\log_{10}(B_T(672)) \leq -1$ appear in black. The red curve (colour online) traces the contour along which $B_T(672) = 1$, such that $B_T(672) > 1$ to the left and $B_T(672) < 1$ to the right of this curve. In Set 1 all treatments eliminate the bacterial population (such that $B_T(672) < 1$), while, for the remaining parameter sets, treatment is effective for sufficiently high α_A and low β_A. Comparison with Figs W–AC, reveals that this is the most effective treatment strategy amongst those considered, not including those which involve continuous debridement. Graphs marked with stars correspond to the parameter sets explored in the main text. Eqs 1–8 were solved using ode15s. See Tables 2 and A for the remaining parameter values.

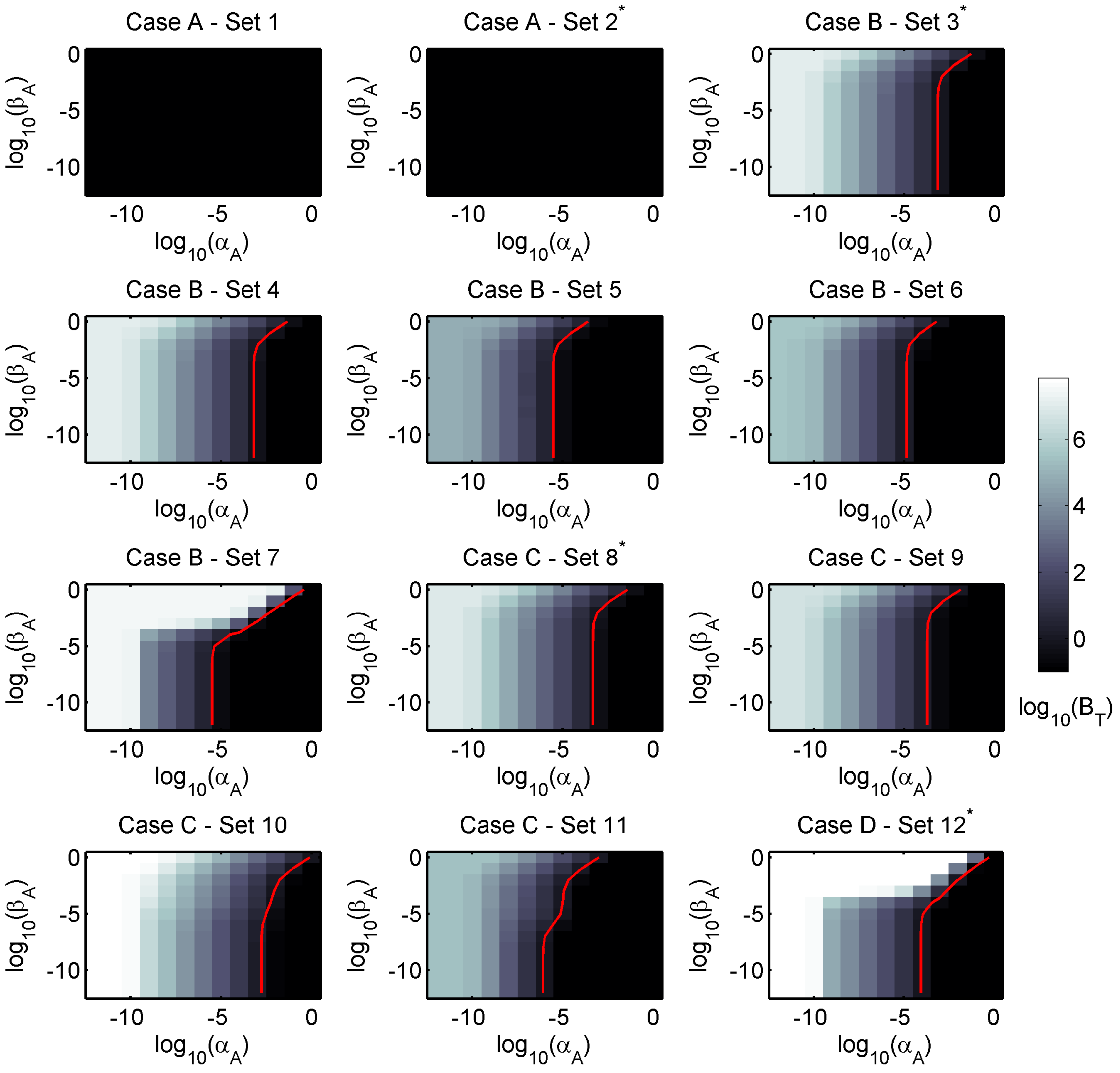

Figure AE: Sensitivity analysis to determine the effect of varying inhibitor binding (α_A) and unbinding (β_A) rates upon the efficacy of a single inhibitor dose combined with continuous debridement, where $A_{F_{init}} = 6.12 \times 10^7$ inhibitors cm^{-3} (the standard value). For each panel, α_A and β_A = 10^{-12}, 10^{-11}, ..., 10^{-1} and 1, where a $\log_{10}$ scale is used on both axes. The value of $\log_{10}(B_T(672))$ is plotted at each point in parameter space, where $B_T(672)$ $(= VB_F(672) + A_r B_B(672))$ is the total number of bacteria after 4 weeks (672 hr). The colour scheme is calibrated to maximise clarity, such that values of $\log_{10}(B_T(672)) \leq -1$ appear in black. The red curve (colour online) traces the contour along which $B_T(672) = 1$, such that $B_T(672) > 1$ to the left and $B_T(672) < 1$ to the right of this curve. In Case A all treatments eliminate the bacterial population (such that $B_T(672) < 1$), while, for the remaining parameter sets, treatment is effective for sufficiently high α_A and low β_A. This treatment strategy is more effective than those involving inhibitor alone, with either inhibitor dose concentration, and those in which inhibitor is combined with regular debridement, with the standard dose concentration, but less effective than those in which inhibitor is combined with regular debridement, with the higher dose concentration (compare Figs W–AD). Graphs marked with stars correspond to the parameter sets explored in the main text. Eqs 1–8 were solved using `ode15s`. See Tables 2 and A for the remaining parameter values.

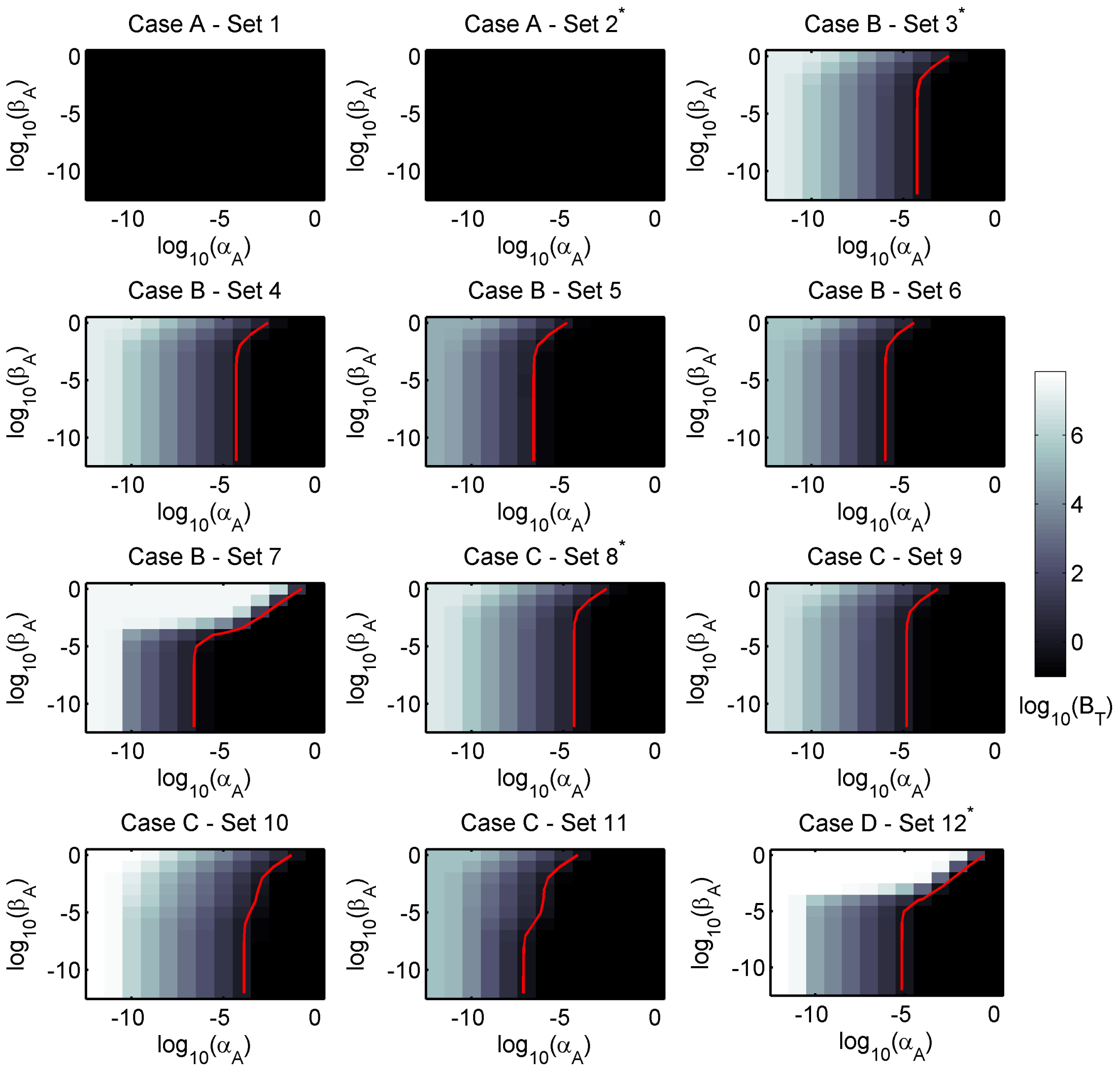

Figure AF: Sensitivity analysis to determine the effect of varying inhibitor binding (α_A) and unbinding (β_A) rates upon the efficacy of a single inhibitor dose combined with continuous debridement, where $A_{F_{init}} = 6.12 \times 10^8$ inhibitors cm^{-3} (10× the standard value). For each panel, α_A and $\beta_A = 10^{-12}, 10^{-11}, \ldots, 10^{-1}$ and 1, where a $\log_{10}$ scale is used on both axes. The value of $\log_{10}(B_T(672))$ is plotted at each point in parameter space, where $B_T(672)$ ($= VB_F(672) + A_r B_B(672)$) is the total number of bacteria after 4 weeks (672 hr). The colour scheme is calibrated to maximise clarity, such that values of $\log_{10}(B_T(672)) \le -1$ appear in black. The red curve (colour online) traces the contour along which $B_T(672) = 1$, such that $B_T(672) > 1$ to the left and $B_T(672) < 1$ to the right of this curve. In Case A all treatments eliminate the bacterial population (such that $B_T(672) < 1$), while, for the remaining parameter sets, treatment is effective for sufficiently high α_A and low β_A. This treatment strategy is the most effective of all those considered (compare Figs W–AE). Graphs marked with stars correspond to the parameter sets explored in the main text. Eqs 1–8 were solved using `ode15s`. See Tables 2 and A for the remaining parameter values.

Table B: Upper and lower bounds of adhesion-related parameters for inhibitors at the standard dose concentration for which treatment is predicted to be successful. For each treatment scenario and parameter set the minimum binding rate, α_A, the maximum unbinding rate, β_A, the minimum inhibitor association constant, α_A/β_A and the maximum ratio of inhibitor and bacterial association constants $(\alpha_{Bac}/\beta_{Bac})/(\alpha_A/\beta_A)$ for which treatment is predicted to be effective in eliminating a bacterial infection within 4 weeks are given. Figures are stated to an accuracy of at most 3 s.f. Where figures are given in parentheses, treatment was predicted to be effective throughout the parameter ranges tested.

Quantity	Value											
	Case A		Case B					Case C				Case D
	Set 1	Set 2	Set 3	Set 4	Set 5	Set 6	Set 7	Set 8	Set 9	Set 10	Set 11	Set 12
Single Inhibitor Dose with Regular Debridement — Standard Dose												
$\min(\alpha_A)$	(10^{-12})	10^{-10}	10^{-2}	10^{-2}	10^{-4}	10^{-3}	10^{-3}	10^{-2}	10^{-2}	10^{-2}	10^{-5}	10^{-2}
$\max(\beta_A)$	(1)	1	1	1	1	1	1	1	1	1	1	1
$\min(\alpha_A/\beta_A)$	(10^{-12})	10^{-7}	1	1	10^{-2}	10^{-1}	10^{-1}	1	1	1	10^{-2}	10^{-1}
$\max(\frac{\alpha_{Bac}/\beta_{Bac}}{\alpha_A/\beta_A})$	(5.14×10^{1})	6.82×10^{-2}	7.53×10^{-2}	2.61×10^{-2}	1.91	9.34×10^{-8}	8.41×10^{-1}	2.82×10^{-2}	3.43×10^{-1}	2.61	1.48×10^{-5}	5.77×10^{-5}
Regular Inhibitor Doses with Regular Debridement — Standard Dose												
$\min(\alpha_A)$	(10^{-12})	10^{-11}	10^{-2}	10^{-2}	10^{-4}	10^{-3}	10^{-3}	10^{-2}	10^{-2}	10^{-2}	10^{-5}	10^{-2}
$\max(\beta_A)$	(1)	1	1	1	1	1	1	1	1	1	1	1
$\min(\alpha_A/\beta_A)$	(10^{-12})	10^{-8}	1	1	10^{-2}	10^{-1}	10^{-2}	1	1	1	10^{-3}	10^{-1}
$\max(\frac{\alpha_{Bac}/\beta_{Bac}}{\alpha_A/\beta_A})$	(5.14×10^{1})	6.82×10^{-1}	7.53×10^{-2}	2.61×10^{-2}	1.91	9.34×10^{-8}	8.41	2.82×10^{-2}	3.43×10^{-1}	2.61	1.48×10^{-4}	5.77×10^{-5}
Single Inhibitor Dose with Continuous Debridement — Standard Dose												
$\min(\alpha_A)$	(10^{-12})	(10^{-12})	10^{-3}	10^{-3}	10^{-5}	10^{-4}	10^{-5}	10^{-3}	10^{-3}	10^{-2}	10^{-6}	10^{-4}
$\max(\beta_A)$	(1)	(1)	1	1	1	1	1	1	1	1	1	1
$\min(\alpha_A/\beta_A)$	(10^{-12})	(10^{-12})	10^{-1}	10^{-1}	10^{-3}	10^{-3}	1	10^{-1}	10^{-1}	1	10^{-3}	1
$\max(\frac{\alpha_{Bac}/\beta_{Bac}}{\alpha_A/\beta_A})$	(5.14×10^{1})	(6.82×10^{3})	7.53×10^{-1}	2.61×10^{-1}	1.91×10^{1}	9.34×10^{-6}	8.41×10^{-2}	2.82×10^{-1}	3.43	2.61	1.48×10^{-4}	5.77×10^{-6}

Table C: Upper and lower bounds of adhesion-related parameters for inhibitors at the higher dose concentration for which treatment is predicted to be successful. For each treatment scenario and parameter set the minimum binding rate, α_A, the maximum unbinding rate, β_A, the minimum inhibitor association constant, α_A/β_A and the maximum ratio of inhibitor and bacterial association constants $(\alpha_{Bac}/\beta_{Bac})/(\alpha_A/\beta_A)$ for which treatment is predicted to be effective in eliminating a bacterial infection within 4 weeks are given. Figures are stated to an accuracy of at most 3 s.f. Where figures are given in parentheses, treatment was predicted to be effective throughout the parameter ranges tested.

Quantity	Value											
	Case A		Case B					Case C				Case D
	Set 1	Set 2	Set 3	Set 4	Set 5	Set 6	Set 7	Set 8	Set 9	Set 10	Set 11	Set 12
Single Inhibitor Dose with Regular Debridement — Concentrated Dose												
$\min(\alpha_A)$	(10^{-12})	10^{-11}	10^{-3}	10^{-3}	10^{-5}	10^{-4}	10^{-4}	10^{-3}	10^{-3}	10^{-3}	10^{-6}	10^{-3}
$\max(\beta_A)$	(1)	1	1	1	1	1	1	1	1	1	1	1
$\min(\alpha_A/\beta_A)$	(10^{-12})	10^{-8}	10^{-1}	10^{-1}	10^{-3}	10^{-2}	10^{-2}	10^{-1}	10^{-2}	10^{-1}	10^{-3}	10^{-2}
$\max(\frac{\alpha_{Bac}/\beta_{Bac}}{\alpha_A/\beta_A})$	(5.14×10^{1})	6.82×10^{-1}	7.53×10^{-1}	2.61×10^{-1}	1.91×10^{1}	9.34×10^{-7}	8.41	2.82×10^{-1}	3.43×10^{1}	2.61×10^{1}	1.48×10^{-4}	5.77×10^{-4}
Regular Inhibitor Doses with Regular Debridement — Concentrated Dose												
$\min(\alpha_A)$	(10^{-12})	10^{-12}	10^{-3}	10^{-3}	10^{-5}	10^{-4}	10^{-4}	10^{-3}	10^{-3}	10^{-3}	10^{-6}	10^{-3}
$\max(\beta_A)$	(1)	1	1	1	1	1	1	1	1	1	1	1
$\min(\alpha_A/\beta_A)$	(10^{-12})	10^{-9}	10^{-1}	10^{-1}	10^{-4}	10^{-2}	10^{-3}	10^{-1}	10^{-2}	10^{-1}	10^{-4}	10^{-2}
$\max(\frac{\alpha_{Bac}/\beta_{Bac}}{\alpha_A/\beta_A})$	(5.14×10^{1})	6.82	7.53×10^{-1}	2.61×10^{-1}	1.91×10^{2}	9.34×10^{-7}	8.41×10^{1}	2.82×10^{-1}	3.43×10^{1}	2.61×10^{1}	1.48×10^{-3}	5.77×10^{-4}
Single Inhibitor Dose with Continuous Debridement — Concentrated Dose												
$\min(\alpha_A)$	(10^{-12})	(10^{-12})	10^{-4}	10^{-4}	10^{-6}	10^{-5}	10^{-6}	10^{-4}	10^{-4}	10^{-3}	10^{-7}	10^{-5}
$\max(\beta_A)$	(1)	(1)	1	1	1	1	1	1	1	1	1	1
$\min(\alpha_A/\beta_A)$	(10^{-12})	(10^{-12})	10^{-2}	10^{-2}	10^{-4}	10^{-4}	10^{-1}	10^{-2}	10^{-3}	10^{-1}	10^{-4}	1
$\max(\frac{\alpha_{Bac}/\beta_{Bac}}{\alpha_A/\beta_A})$	(5.14×10^{1})	(6.82×10^{3})	7.53	2.61	1.91×10^{2}	9.34×10^{-5}	8.41×10^{-1}	2.82	3.43×10^{2}	2.61×10^{1}	1.48×10^{-3}	5.77×10^{-6}
