# Supplementary material for: Predictive modelling of a novel anti-adhesion therapy to combat bacterial colonisation of burn wounds
Source: PLoS Comput Biol. 2018 May 3;14(5):e1006071. doi: 10.1371/journal.pcbi.1006071 (PMC5933687; doi:10.1371/journal.pcbi.1006071)
Supplement: S3 Supporting Information — (PDF) [file pcbi.1006071.s003.pdf]

Predictive modelling of a novel anti-adhesion therapy to combat bacterial colonisation of burn wounds
PLOS Computational Biology
S3 Supporting Information

Paul A. Roberts*[1,2], Ryan M. Huebinger[3], Emma Keen[2], Anne-Marie Krachler[4] and Sara Jabbari[1,2]

[1]School of Mathematics, University of Birmingham, Edgbaston, Birmingham, United Kingdom
[2]Institute of Microbiology and Infection, School of Biosciences, University of Birmingham, Edgbaston, Birmingham, United Kingdom
[3]Department of Surgery, University of Texas Southwestern Medical Center, Dallas, Texas, United States of America
[4]Department of Microbiology and Molecular Genetics, University of Texas McGovern Medical School at Houston, Houston, Texas, United States of America

Steady-state analysis

Steady-state analyses of Eqs 1–6, in the absence of clearance, with and without a single dose of inhibitors were performed using Maple. Clearance was neglected since leakage of fluid from the wound only occurs in the first 24 hours. It was found that the system has two physically realistic steady-states in both the untreated and single inhibitor dose scenarios for all 12 parameter sets. In the untreated scenario, the first steady-state, $(B^*_{F_1}, B^*_{B_1}) = (0,0)$, corresponds to the complete absence of bacteria and can be classified as a saddle-node in all cases, with an unstable manifold directed into the positive quadrant. For the second steady-state, $(B^*_{F_2}, B^*_{B_2})$, we have that $B^*_{F_2} > 0$ and $B^*_{B_2} > 0$ in all cases. Depending upon the parameter set, this steady-state takes the form of either a stable improper node or a stable spiral (see Table A in S2 Supporting Information).

In the treated scenario, since we are neglecting clearance, the total number of inhibitors ($A_T = VA_F(t) + A_rA_B(t) = VA_{F_{init}}$) is conserved. Therefore, we may substitute for $A_B(t)$ as $A_B(t) = h(A_{F_{init}} - A_F(t))$ into Eqs 1–4, reducing the problem to the following three-dimensional system:

$$\frac{\mathrm{d}B_F}{\mathrm{d}t} = r_F B_F\left(1-\frac{B_F}{K_F}\right) + (1-\eta(E))H(K_B - B_B)\frac{r_B}{h}B_B\left(1-\frac{B_B}{K_B}\right) - \alpha_{Bac}A_rB_FE + \frac{\beta_{Bac}}{h}B_B, \tag{A}$$

$$\frac{\mathrm{d}B_B}{\mathrm{d}t} = (1+(\eta(E)-1)H(K_B-B_B))r_BB_B\left(1-\frac{B_B}{K_B}\right) + \alpha_{Bac}VB_FE - \beta_{Bac}B_B - \delta_BB_B, \tag{B}$$

$$\frac{\mathrm{d}A_F}{\mathrm{d}t} = -\alpha_AA_rA_FE + \beta_A(A_{F_{init}} - A_F). \tag{C}$$

This system has two steady-states; the first, $(B^*_{F_1}, B^*_{B_1}, A^*_{F_1}) = (0,0,A^*_{F_1})$, where $A^*_{F_1} > 0$, corresponds to the complete absence of bacteria and can be classified as a saddle node (with either one or two unstable manifolds) in all

*Corresponding author
E-mail address: p.a.roberts@univ.oxon.org (PAR)

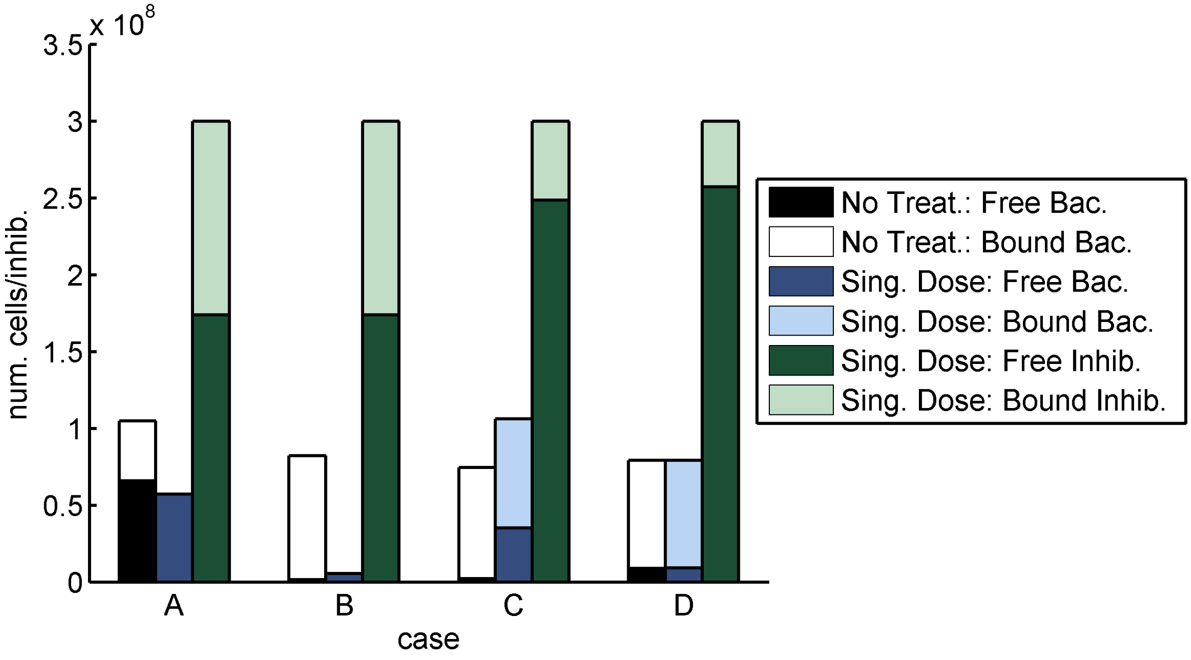

Figure A: Steady-state solutions to Eqs 1–6 with and without a single dose of inhibitor. Three stacked bars are plotted for each case: the first bar shows the number of free and bound bacteria, $\hat{B}^*_{F_2} = VB^*_{F_2}$ and $\hat{B}^*_{B_2} = A_r B^*_{B_2}$, at steady-state in the untreated scenario; the second gives the number of free and bound bacteria at steady-state in the single inhibitor dose scenario, and the third shows the number of free and bound inhibitors, $\hat{A}^*_{F_2} = VA^*_{F_2}$ and $\hat{A}^*_{B_2} = A_r A^*_{B_2}$, at steady-state in the single inhibitor dose scenario. The combined height of each stacked bar gives the total number of bacteria or inhibitors, $B^*_{T_2} = \hat{B}^*_{F_2} + \hat{B}^*_{B_2}$ and $A_{F_{init}} = \hat{A}^*_{F_2} + \hat{A}^*_{B_2}$. Treatment results in a decrease in $B^*_{T_2}$ in Cases A and B, an increase in $B^*_{T_2}$ in Case C and has little effect on $B^*_{T_2}$ in Case D. The ratio of free to bound inhibitors is similar in Cases A and B, and varies in Cases C and D. Steady-state solutions were obtained by solving Eqs 1–8 using ode15s, allowing the system to evolve until it reached steady-state. The problem was solved in the absence of clearance, such that $\tilde{\psi}_{Bac} = 0$ and $\tilde{\psi}_A = 0$. See Tables 1 and 2 for the remaining parameter values.

cases. For the second steady-state, $(B^*_{F_2}, B^*_{B_2}, A^*_{F_2})$, we have that $B^*_{F_2} > 0$, $B^*_{B_2} > 0$ and $A^*_{F_2} > 0$ in all cases, though $B^*_{B_2}$ is tiny by comparison with $B^*_{F_2}$ and $A^*_{F_2}$ for Cases A and B (see Fig A, and Fig C in S2 Supporting Information, where $B^*_{B_2}$ is too small to be visible in Cases A and B). This steady-state takes the form of a stable improper node (three non-identical real negative eigenvalues) for all parameter sets, except Set 8, where it is a stable spiral (one real negative eigenvalue and a pair of complex conjugate eigenvalues with negative real part).

The discovery that the stable steady-state may take the form of a node or a spiral may be of value in identifying the most accurate parameter set, if it were possible to take highly resolved experimental measurements, so as to detect, or rule out, oscillations in the bacterial population size. We note, however, that experimental error might render observations of oscillations difficult.

The steady-state solutions in the untreated and single inhibitor dose scenarios are summarised in Fig A, and Fig C in S2 Supporting Information. Treatment results in a decrease in the total number of bacteria in Cases A and B, as might be expected from the experimental results (see Fig 2); however, it results in an increase in Case C and has little effect on the total population size in Case D. These latter two results are unexpected, suggesting that in some situations treatment with inhibitor could be detrimental in the long-term. We discuss this further in the numerical solutions section of the main text. The ratio of free inhibitors to bound inhibitors is consistent across all parameter sets in Cases A and B, but varies across parameter sets in Cases C and D, with a lower proportion of bound inhibitors in Cases C and D.
