# Supplementary material for: Predictive modelling of a novel anti-adhesion therapy to combat bacterial colonisation of burn wounds
Source: PLoS Comput Biol. 2018 May 3;14(5):e1006071. doi: 10.1371/journal.pcbi.1006071 (PMC5933687; doi:10.1371/journal.pcbi.1006071)
Supplement: S4 Supporting Information — (PDF) [file pcbi.1006071.s004.pdf]

# Predictive modelling of a novel anti-adhesion therapy to combat bacterial colonisation of burn wounds
# PLOS Computational Biology
# S4 Supporting Information

Paul A. Roberts*[1,2], Ryan M. Huebinger[3], Emma Keen[2], Anne-Marie Krachler[4] and Sara Jabbari[1,2]

[1]School of Mathematics, University of Birmingham, Edgbaston, Birmingham, United Kingdom
[2]Institute of Microbiology and Infection, School of Biosciences, University of Birmingham, Edgbaston, Birmingham, United Kingdom
[3]Department of Surgery, University of Texas Southwestern Medical Center, Dallas, Texas, United States of America
[4]Department of Microbiology and Molecular Genetics, University of Texas McGovern Medical School at Houston, Houston, Texas, United States of America

## Numerical solutions — additional details

Taking Cases A–D in turn, we note the distinguishing features of each case, explain the solution behaviour in the untreated and single inhibitor dose scenarios, and investigate other treatment strategies.

### Case A

Distinguishing features of the model predictions:

- The total numbers of bacteria in both the untreated and single inhibitor dose scenarios are essentially monotone increasing functions of time, achieving their steady-state values earlier than in the other cases;
- All treatments are effective in reducing the number of bacteria;
- All treatments except for single and regular inhibitor doses eliminate the bacterial population (such that $B_T(t) < 1$) by $t = 2000$ hr.

Explanation for the effect of treatment with inhibitors:
The evolution in the total number of bacteria, $B_T(t)$, is qualitatively similar for both sets in the untreated scenario and similarly in the single inhibitor dose scenario (see Fig 5, and Fig D in S2 Supporting Information); however, interestingly, the parameter sets achieve this total by different means in the untreated scenario. In Set 1, the majority of bacteria are predicted to be free in the absence of treatment, while in Set 2, both free and bound bacteria are present in similar numbers (see Fig 6, and Fig E in S2 Supporting Information). Thus, Set 2 is more biologically realistic than Set 1, since we would expect a significant number of bacteria (at least $O(E_{init} \times 10^{-1})$) to bind to the host in the absence of inhibitor.

*Corresponding author
E-mail address: p.a.roberts@univ.oxon.org (PAR)

Sensitivity analysis shows that $B_T(t)$ is most sensitive to the free carrying capacity, $K_F$, in both the untreated and single inhibitor dose scenarios, showing no significant sensitivity to any other fitted parameter in the single inhibitor dose scenario (see Figs O and P in S2 Supporting Information). This parameter is significant in the latter scenario since the majority of bacteria are in the free compartment when treatment is applied, achieving their carrying capacities. This also suggests that regular and continuous debridement should both be effective in reducing the bacterial population size.

Exploring other treatment strategies:
In both Sets 1 and 2, the rate constant of bacterial unbinding is high (see Table 1, and Table A in S2 Supporting Information), so that even when inhibitors are not used, regular and continuous debridement are effective in eliminating bacteria. Sensitivity analysis of treatment strategies 3–8 shows that regular inhibitor dosing is sensitive to $K_F$, similar to treatment with a single inhibitor dose, while regular debridement is sensitive to $r_F$, $r_B$, $\alpha_{Bac}$, $\beta_{Bac}$ and $\tilde{\psi}_{Bac}$, parameter variation resulting in an increase in $B_T(t)$ in all those cases where the change is significant. The remaining treatment strategies are almost entirely insensitive to variation in the fitted parameters (Figs Q–V in S2 Supporting Information) and hence relatively robust.

**Case B**

Distinguishing features of the model predictions:

- Single and regular inhibitor doses, continuous debridement and a single inhibitor dose with continuous debridement all result in a long-term reduction in the total number of bacteria;
- The use of regular inhibitor doses with regular debridement is consistently effective in significantly reducing (parameter sets 3, 4, 6 and 7) or removing (parameter set 5) the bacterial population;
- Regular debridement and a single inhibitor dose with regular debridement are not consistently effective in reducing the bacterial burden.

Explanation for the effect of treatment with inhibitors:
The evolution in the total number of bacteria, $B_T(t)$, is qualitatively similar for each set in the untreated scenario and similarly in the single inhibitor dose scenario (see Fig 5, and Fig D in S2 Supporting Information). In the untreated scenario, $B_T(t)$ achieves an early maximum at about 100 hr, before settling to a reduced steady-state. The same behaviour is present in Cases C and D (see Fig 5, and Fig D in S2 Supporting Information). It can be seen from Fig 6 and Fig E in S2 Supporting Information that the drop in $B_T(t)$ is caused by a drop in $B_F(t)$, the bound bacterial population size being essentially monotone increasing. Examination of the terms in Eq 1 in Cases B–D reveals that the early growth in free bacterial numbers is due to the contribution of daughter cells from the bound compartment, while the subsequent reduction in numbers occurs as the bound bacteria reach or exceed their carrying capacity, $K_B$ (see Fig G in S2 Supporting Information). At this point the supply of bacteria to the free compartment from the bound compartment is greatly diminished or ceases, and the number of free bacteria drops through binding to the surface and (with the exception of Case C, Set 10) death, free bacteria having exceeded their carrying capacity, $K_F$.

Bound bacteria are predicted to be present in significant numbers for all parameter sets in Case B, outnumbering free bacteria at steady-state in parameter sets 3–5 (see Fig 6, and Fig E in S2 Supporting Information).

Sensitivity analysis of the single inhibitor dose scenario shows that $B_T(t)$ is most sensitive to $K_F$, with significant sensitivity to $\alpha_{Bac}$, $\delta_B$ and $\alpha_A$ in Sets 3 and 4 (see Fig P in S2 Supporting Information). The free carrying capacity is significant since most bacteria are in the free compartment when treatment is applied. By contrast, $B_T(t)$ is most sensitive to $K_B$ in the untreated scenario (see Fig O in S2 Supporting Information).

Exploring other treatment strategies:
Combining inhibitor with continuous debridement is more effective than inhibitor alone, in all cases except Set 7, where the addition of continuous debridement results in an increased $B_T(t)$ (see Fig N in S2 Supporting Information). This increase may be explained by the significant contribution made to $B_B(t)$ by logistic growth in Set 7, in the single dose scenario, whereas binding plays a more significant role in Sets 3–6 (see Fig J in S2 Supporting Information).

By removing free inhibitors before they have finished binding, more bound daughter cells may colonize the surface, increasing the bound population size, and hence $B_T(t)$, above that in the single inhibitor dose scenario.

The regular inhibitor dosing scenario is more sensitive to changes in the parameters than the single inhibitor scenario, though it does not show a consistently strong sensitivity to any one parameter (Fig Q in S2 Supporting Information). The regular debridement scenario is most sensitive to $K_B$ (Fig R in S2 Supporting Information), consistent with the observation that regular debridement is more effective when combined with inhibitor, which may have a similar effect to reducing $K_B$, while treatments combining inhibitor with regular debridement are most sensitive to $r_B$ in general (Figs S and T in S2 Supporting Information). Treatments involving continuous debridement are relatively insensitive to parameter changes for the most part, showing no consistent sensitivity to any one parameter (Figs U and V in S2 Supporting Information).

**Case C**

Distinguishing features of the model predictions:

- Single, and in some cases (parameter sets 8 and 10) regular, inhibitor doses increase the total bacterial population size.

Explanation for the effect of treatment with inhibitors:
The evolution in the total number of bacteria, $B_T(t)$, is qualitatively similar for each set in the untreated scenario and similarly in the single inhibitor dose scenario (see Fig 5, and Fig D in S2 Supporting Information).

Set 10 is peculiar in that a single inhibitor dose increases the numbers of both bound and free bacteria, despite the fact that bound bacteria exceed their carrying capacity in the absence of treatment (compare Figs E and F in S2 Supporting Information and see also Fig C in S2 Supporting Information). Treatment causes an increase in the number of free bacteria by reducing the number of free binding sites, which reduces the rate at which free bacteria bind to the surface, this being the main sink term in the untreated scenario (see Figs G and I in S2 Supporting Information). As the solution approaches steady-state, the rate of binding of free bacteria comes to exceed that in the untreated scenario, despite the decreased number of binding sites, since the number of free bacteria is now almost three orders of magnitude larger than in the untreated scenario. As a consequence of the increased rate of binding of free bacteria, the steady-state number of bound bacteria exceeds that in the untreated scenario.

Sensitivity analysis shows that the system is most sensitive to $K_B$ in the untreated scenario (Fig O in S2 Supporting Information), while in the single inhibitor dose scenario, parameter sets show significant sensitivity to $r_F$, $r_B$, $K_F$ and $K_B$ (Fig P in S2 Supporting Information). This suggests that, in Case C, treatment efficacy will depend to a large extend upon the bacterial population size that can be supported by the bound and free compartments and the rate at which the bacterial species can reproduce or die. Counter-intuitively, an increase in $r_F$ (the intrinsic growth rate of free bacteria) typically results in a decrease in $B_T(t)$. This is because the logistic growth term acts mainly as a death term, removing free bacteria from the exudate as their population size is pushed above its carrying capacity by the flux of daughter cells from the bound compartment.

Exploring other treatment strategies:
Regular inhibitor dosing is most sensitive to $r_F$, $r_B$, $K_F$, $K_B$, $\alpha_{Bac}$, $\delta_B$, $\alpha_A$ and $\beta_A$ (Fig Q in S2 Supporting Information). Treatments involving regular debridement are generally most sensitive to $K_B$ (Figs R–T in S2 Supporting Information), while those involving continuous debridement are not consistently sensitive to any one parameter (Figs U and V in S2 Supporting Information).

**Case D**

Distinguishing features of the model predictions:

- A single inhibitor dose causes the total bacterial population size to temporarily exceed that without treatment and is close to that without treatment at steady-state, though it matches the experimental result over the first six days;

- All treatments are ineffective, the long-term bacterial population size being close to that without treatment in all cases.

Explanation for the effect of treatment with inhibitors:
In the untreated scenario, $B_T(t)$ evolves in a qualitatively similar way to Cases B and C, while application of a single inhibitor dose has little effect on the steady-state value of $B_T(t)$ (see Fig 5, and Fig D in S2 Supporting Information). The number of bound bacteria exceeds that of free bacteria in the untreated scenario (see Fig 6, and Fig E in S2 Supporting Information).

In both the untreated and treated scenarios the growth in the population size of free bacteria is due to the flux of bound daughter cells as in Cases B and C (see Figs G and I in S2 Supporting Information). However, unlike in Cases A–C (with the exception of Set 7), the increase in the number of bound bacteria is primarily due to their logistic growth, rather than the binding of free bacteria (see Figs H and J in S2 Supporting Information).

Sensitivity analysis shows that the system is most sensitive to $K_F$ and $K_B$ in both the untreated and single inhibitor dose scenarios (see Figs O and P in S2 Supporting Information). This is intuitive, given that $B_F(t)$ and $B_B(t)$ take values close to carrying capacity at steady-state in both scenarios.

Exploring other treatment strategies:
Treatment involving regular inhibitor doses, regular debridement or continuous debridement are all ineffective in Case D (see Fig 7, and Figs M and N in S2 Supporting Information). Regular inhibitor dosing shows greatest sensitivity to $r_F$ and $K_F$, while treatments involving regular or continuous debridement show significant sensitivity to $K_B$ only (Figs Q–V in S2 Supporting Information).

### Inhibitor sensitivity analysis

Tables B and C in S2 Supporting Information show the minimum value of $\alpha_A$, the maximum value of $\beta_A$, the minimum value of $\alpha_A/\beta_A$ and the maximum ratio of bacterial to inhibitor association constants, $(\alpha_{Bac}/\beta_{Bac})/(\alpha_A/\beta_A)$, predicted to be required for each treatment to eliminate the bacterial population for each parameter set, for both standard and higher concentration inhibitor doses. It can be seen that $\alpha_A$ must be at least $10^{-2}$ hr$^{-1}$ sites$^{-1}$ in order for each of the treatments to eliminate the bacterial burden for all parameter sets when standard inhibitor doses are used, and that a value of $10^{-3}$ hr$^{-1}$ sites$^{-1}$ will suffice where higher concentration doses are used.

The model predicts that the inhibitor association constant, $\alpha_A/\beta_A$, must take a minimum value of 1 sites$^{-1}$ in order for treatment to eliminate the bacterial burden across all parameter sets for each of the treatments in the standard dose scenario and for single inhibitor with continuous debridement in the higher concentration dose scenario, while a value of $10^{-1}$ sites$^{-1}$ suffices for single and regular inhibitor with regular debridement in the higher concentration dose scenario. Lastly, in order for treatment to eliminate the bacterial population, the ratio of bacterial to inhibitor association constants must be no more than $\approx 10^{-7}$ in the single and regular inhibitor with regular debridement scenarios with standard doses, $\approx 10^{-6}$ in the single and regular inhibitor with regular debridement scenarios with higher concentration doses, and $\approx 6 \times 10^{-6}$ in the single inhibitor with continuous debridement scenario with standard or higher concentration doses. Thus, the inhibitor association constant must be 6–7 orders of magnitude larger than that of the bacteria in order for treatment to eliminate the bacterial population in all cases, though the association constant can be much lower in many parameter sets (e.g. in Sets 5, 9 and 10, the inhibitor association constant can be $O(10)$–$O(100)$ times smaller than the bacteria association constant for all three treatments in the higher concentration dose scenario).
